# Disentangling Timescales of Molecular Kinetics with spFRET using ALEX-FCS

**DOI:** 10.1007/s10895-025-04187-0

**Published:** 2025-02-17

**Authors:** Jeremy Ernst, Aditya Sane, John van Noort

**Affiliations:** Biological and Soft Matter Physics, Huygens-Kamerlingh Onnes Laboratory, Leiden University, Niels Bohrweg 2, 2333 CA Leiden, The Netherlands

**Keywords:** FRET, FCS, ALEX, Conformational dynamics

## Abstract

Single-pair Förster resonance energy transfer (spFRET) probes the dynamics of molecular structures with (sub-)nanometer accuracy. When combined with fluorescence correlation spectroscopy (FCS), diffusion times and conformation lifetimes can be obtained. Alternating excitation (ALEX) further complements spFRET measurements on freely diffusing molecules, allowing for burst analysis, which can be used to reduce background signal without significant changes to the experimental setup. ALEX is particularly useful for extracting conformational dynamics, but extracting small differences in FRET levels and/or diffusion times can still be difficult for multi-species samples with fast or slow transition rates. Though the combination of spFRET, FCS and ALEX can help to constrain the fits of correlation curves, a rigorous analysis of the range of lifetimes that can be probed with a combination of these methods is lacking. Here, we simulated spFRET-ALEX-FCS experiments of molecules with two conformations that differ both in FRET levels and in diffusion coefficients, representative of fully wrapped and partially unwrapped nucleosomes. We show that we can distinguish small changes in the diffusion coefficient and that burst selection yields accurate lifetimes ranging from 100 us to 100 ms. The simulations provide a framework that can be expanded for more complex systems having a larger number of conformational states, variable stoichiometries from binding interactions and/or other excitation schemes.

**Supplementary Information:**

The online version contains supplementary material available at 10.1007/s10895-025-04187-0.

## Introduction

Single-molecule fluorescence techniques provide a unique view into the dynamic properties of biological systems. While complete Angstrom resolution structures can be obtained with X-ray crystallography [1], nuclear magnetic resonance (NMR) [2] or cryogenic electron microscopy (cryo-EM) [3, 4], single-pair Förster resonance energy transfer (spFRET) excels in probing inter-molecular distances down to nanoseconds with sub-nanometer accuracy and under physiological conditions. This makes it possible to connect the dynamics of conformational states to their biological function [5, 6]. In FRET, energy is transferred non-radiatively from a donor to an acceptor fluorophore and the transfer efficiency depends on the distance between the two [7, 8]. Labeling and measurements at the single-molecule scale can be done *in vivo* and *in vitro*, and a wide range of biological applications have revealed conformational changes and molecular interactions with high temporal and spatial accuracy.

A convenient way to reach high temporal accuracy and to minimize interference of background fluorescence is to combine spFRET with fluorescence correlation spectroscopy (FCS) [9], first introduced by Elson and Magde [10]. Fluctuations of the fluorescence signal are quantified by (cross-)correlation, revealing the dynamics of conformation changes and interactions between different molecules. In addition, the hydrodynamic radii of the molecules can be calculated from their diffusion time through the excitation volume. It allows for tether-free experiments on fluorescent molecules and can be used *in vivo* in combination with fluorescent proteins or orthogonal labeling chemistry [11–16]. Theoretical frameworks for the effect of changes in FRET state on the correlation function have been described. [17, 18].

Alternatively, two-color fluorescence cross-correlation spectroscopy (FCCS) can directly measure the interactions between molecules, [19, 20] and does not depend on the energy transfer of the two fluorophores. The two techniques are not mutually exclusive and can be combined in the same experiment.

To discriminate the absence of FRET due to inter-molecular distances larger than the Förster radius from the absence of FRET due to the inactivity of the acceptor fluorophore, Alternating Laser EXcitation (ALEX) was introduced, where both fluorophores are excited alternatingly [21]. This is especially important for spFRET, as fluorophore bleaching, blinking and/or incomplete labeling efficiency can be confused with extended conformations. By alternating the excitation wavelength with a period of several microseconds, the stoichiometry of fluorescent bursts can be recovered simultaneously with the FRET efficiency. In sufficiently diluted solutions, each burst represents a single molecule diffusing through the focus. Burst population analysis then allows for separating different FRET states, as well as the kinetic constants of transitions between them [22, 23]. More recently, Pulsed-Interleaved Excitation (PIE) [24, 25] and similar nsALEX [26] was introduced, resolving sub-microsecond phenomena [27]. Both methods use the higher laser modulation frequency of pulsed lasers, often in combination with time-correlated single photon counting (TCSPC) [28] or multi-parameter fluorescence detection (MFD) [29] to extract fluorescence lifetime information of the measured populations. This allows for the photons to be correlated not only spectrally, but also by fluorescence lifetime [30]. There now exist extensive analysis frameworks for these methods [31–33] as well as their combination with spFRET [34, 35].

The experimental set-up for FCS, ALEX and PIE are very similar. Using a confocal microscope equipped with intensity-modulated lasers and single-photon detectors, (sub-)nanomolar sample concentrations are measured with single-molecule precision. The resulting data set of photon arrival times can be processed using correlation analysis and/or burst analysis. However, ALEX-spFRET and correlation analysis methods are not always combined in a complementary fashion [36], missing an opportunity to maximally constrain fitting parameters and to optimize their accuracy. In this work, we provide a simple and intuitive combination of correlation- and burst analysis methods for extracting physical parameters from ALEX/PIE spFRET measurements.

Data analysis for combined FCS and ALEX is not trivial, as the timescales that define the conformational changes and diffusion may (partially) overlap with those of the excitation scheme. In addition, the limited number of photons complicates the accurate assignment of bursts. While overviews of parameters that can accurately be probed with spFRET methods are available [37, 38], a comprehensive analysis of how changes in FRET efficiency due to dynamic structural changes are dependent on changes in particle size, conformational lifetimes and measured fluorescent signal intensity is missing. Here we use simulations to explore the limits of FCS [39, 40], PIE [29], and spFRET experiments [41].

One application that has greatly benefited from spFRET methods is research into the dynamics of DNA and chromatin structure. Nucleosome folding, breathing and disassembly dynamics have previously been extensively studied [42, 43], as well as DNA-protein [44, 45] and nucleosome-protein [46, 47] interactions. These studies have yielded a wide range of relevant timescales, ranging from microseconds to seconds. It is, therefore, important to understand the limitations of different spFRET methods for obtaining accurate parameters. As an example application, we present simulations of FRET-labeled nucleosomes and chromatin fibers and aim to separate local conformational changes from changes in the global structure of the chromatin fiber [48, 49].

**Fig. 1:**
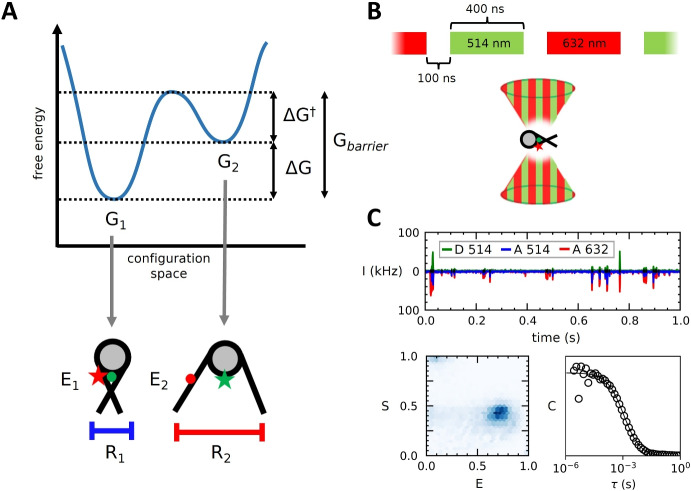
**Nucleosomes were simulated as a two-state system and excited using ALternating Laser Excitation (ALEX). Emitted photons were analyzed using a combined burst- and correlation analysis approach.** **A)** Transitions between states of a two-state molecule are defined by $\Delta G$ and $\Delta G^{\dag }$. Each state represents a conformational state of the nucleosome, with corresponding FRET value *E* and hydrodynamic radius *R*. **B)** Simulated nucleosomes were excited with ALEX. Pulse lengths of the donor (514 nm) and acceptor (632 nm) excitation laser were 400 ns, with 100 ns between. **C)** Photons emitted by the simulated nucleosomes (top) were processed using a combined burst- (bottom, left) and correlation analysis (bottom, right)

## Results

To untangle the various time scales in a typical spFRET experiment, we performed extensive simulations that included diffusion dynamics, conformational dynamics, and experimental timing settings, as well as the geometry of the molecules and the optics. A detailed description of the implementation of these simulations is provided in supplementary material. First, we considered conformational dynamics of a molecule that alternates between two conformational states. We used an energy landscape with two local energy minima, $G_{1}$ and $G_{2}$ and computed a Markov chain of transition events for the transitions between conformational states. Figure 1A shows a schematic overview of this model. The transition rate from state 1 to state 2 depends on the difference in free energy between the two states $\Delta G$, as well as the height of the energy barrier $\Delta G^{\dag }$. $G_{barrier}$ is the sum of these two terms. Transitions from state 2 to state 1 only depend on $\Delta G^{\dag }$.

Using these parameters, trajectories of freely diffusing nucleosomes were generated using Brownian dynamics. In these simulations, the conformational state of each molecule was calculated over time according to the two-state model of its free energy landscape. Each state *i* was then assigned a hydrodynamic radius $R_{i}$ and FRET efficiency $E_{i}$, the first of which was used to calculate the distance traveled by the molecule per simulation time step according to Eq. 7. These nucleosomes were then excited with an ALEX beam, as shown in Fig. 1B. Emitted photons were separated by excitation and emission wavelengths (Fig. 1C, top). FRET values *E* and stoichiometries *S* were calculated for each burst (Fig. 1C, bottom left) and specific burst populations were then analyzed using correlation analysis (Fig. 1C, bottom right).

Unless specified otherwise, all simulations in this section were performed using default parameters representative of settings in our setup and experiments on nucleosomes or chromatin fibers [48, 50]. The simulated time was 15 minutes. FRET values were $E_{1}=0.8$ and $E_{2}=0.1$. $\Delta G= 1.5\, k_{B}T$, corresponding to an equilibrium constant $K = k_{21}/k_{12} = 4.5$. When simulating free diffusion, five particles were enclosed in a box, with dimensions corresponding to a concentration of 50 pM. For these experiments, the laser excitation intensity was adjusted for donor excitation at 514 nm to a fluorescence intensity $I_{0} = 150$ kHz, and for acceptor excitation at 632 nm $I_{0} = 100$ kHz was used.

**Fig. 2:**
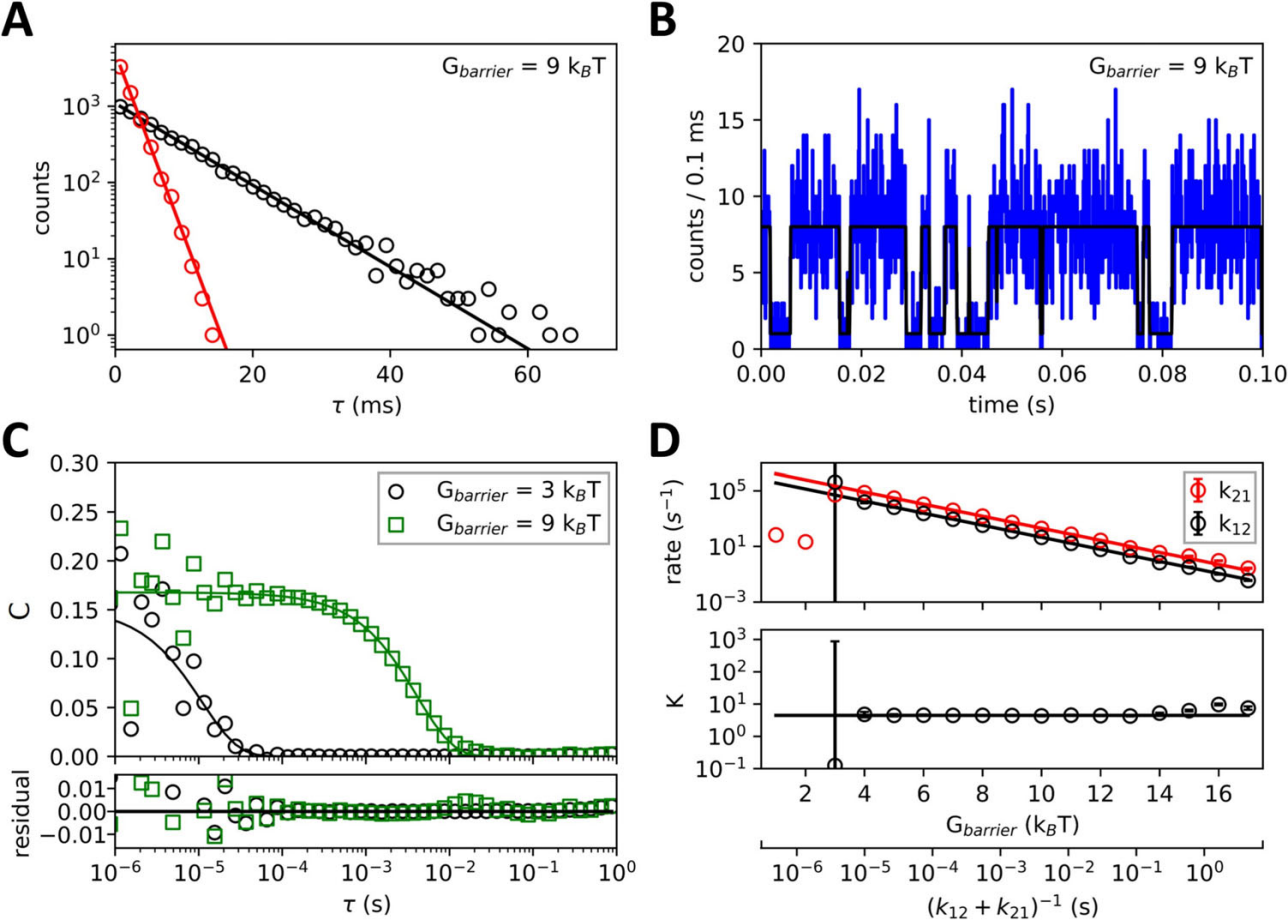
**The conformational state lifetimes and kinetic constants of a two-state system can be extracted down to 10 us by single-pair FRET measurements.** **A)** Distribution of lifetimes for $G_{barrier}=9\, k_{B}T$. Black represents the lowest energy state, and red is the higher energy state. **B)** Time trace of an immobile particle undergoing conformational dynamics with $G_{barrier}=9\, k_{B}T$ (corresponding to $k_{12}=1.2\cdot 10^{2}\,s^{-1}, k_{21}=5.5\cdot 10^{2}\,s^{-1}$). The black line is the simulated intensity and is used as an input to generate the emitted photon distribution, plotted in blue. The bin size is 0.1 ms. **C)** Correlation functions calculated from the FRET photons for $G_{barrier}=9\, k_{B}T$ (green squares) and $3\, k_{B}T$ (corresponding to $k_{12}=5.0\cdot 10^{4}\,s^{-1}, k_{21}=2.2\cdot 10^{5}\,s^{-1}$, black circles). **D)** Fitted transition rates calculated for increasing $G_{barrier}$ show the lower and upper limit of detectable timescales. Errors in *k* could not be estimated for $G_{barrier} < 3\, k_{B}T$. $\Delta G=1.5\, k_{B}T$ for all figures

### Limits of Correlation Analysis for Quantifying Conformational Dynamics with spFRET

We first quantified the limits of determining a molecule’s conformational dynamics through single-molecule FRET correlation analysis using immobile particles with continuous donor excitation $I_{0} = 100$ kHz for 60 s. An extensive framework for FRET based correlation analysis is provided in the Methods section, which reduces to Eq. 27 when there is no diffusion. The only timescales then present are the conformational state lifetimes.

Figure 2A shows the distribution of lifetimes obtained for $G_{barrier}=9\; k_{B}T$. The lines are the expected counts for Eq. 5 multiplied by the number of observed instances of each state throughout the measurement. The simulated intensity time trace (black) and the corresponding FRET photons (blue) of these states are shown in Fig. 2B. The observed fluctuations in intensity are due to the changes between FRET states $E_{1}$ and $E_{2}$. For $G_{barrier}=9\, k_{B}T$ the transitions are observable by eye, while for $G_{barrier}=3\, k_{B}T$ (shown in Fig. S1) these transitions are no longer distinguishable. As lifetimes decrease with smaller $G_{barrier}$, transitions are faster and we need to bin over shorter intervals to observe discrete transitions. This leads to a larger contribution of the shot noise in the time trace, which scales as $\sqrt{I}$, making it more difficult to identify transitions. Figure 2C shows the auto-correlation functions of these measurements. Transition rates were extracted by fitting the auto-correlation curve to Eq. 27.

To gain insight into the fundamental limits of this approach, we simulated multiple experiments with increasing $G_{barrier}$, but with fixed $\Delta G= 1.5\, k_{B}T$, shown in Fig. 2D. The corresponding kinetic relaxation time $\tau _{K} = (k_{12}+k_{21})^{-1}$ calculated from Eq. 2 is shown beneath. We observed three regimes: short lifetimes ($k > 10^{5}\,s^{-1}$) where transition rates could not be determined due to shot noise, intermediate lifetimes ($10^{5}\,s^{-1}> k > 1\,s^{-1}$) where correlation analysis could accurately determine the particle’s transition rates, and long lifetimes ($k < 1\,s^{-1}$) where transition rates could not be determined due to the limited measurement time.

While these regimes hold in general, the specific values of *k* that can accurately be determined depend on excitation intensity, measurement time, and absolute differences in FRET state. Shorter lifetimes could be probed by increasing excitation intensity to reduce the relative effect of shot noise. In practice, this would, however, also increase the rate of bleaching and other photo-physical effects. Similarly, longer lifetimes could be probed by increasing measurement time but would also be limited by bleaching.

**Fig. 3:**
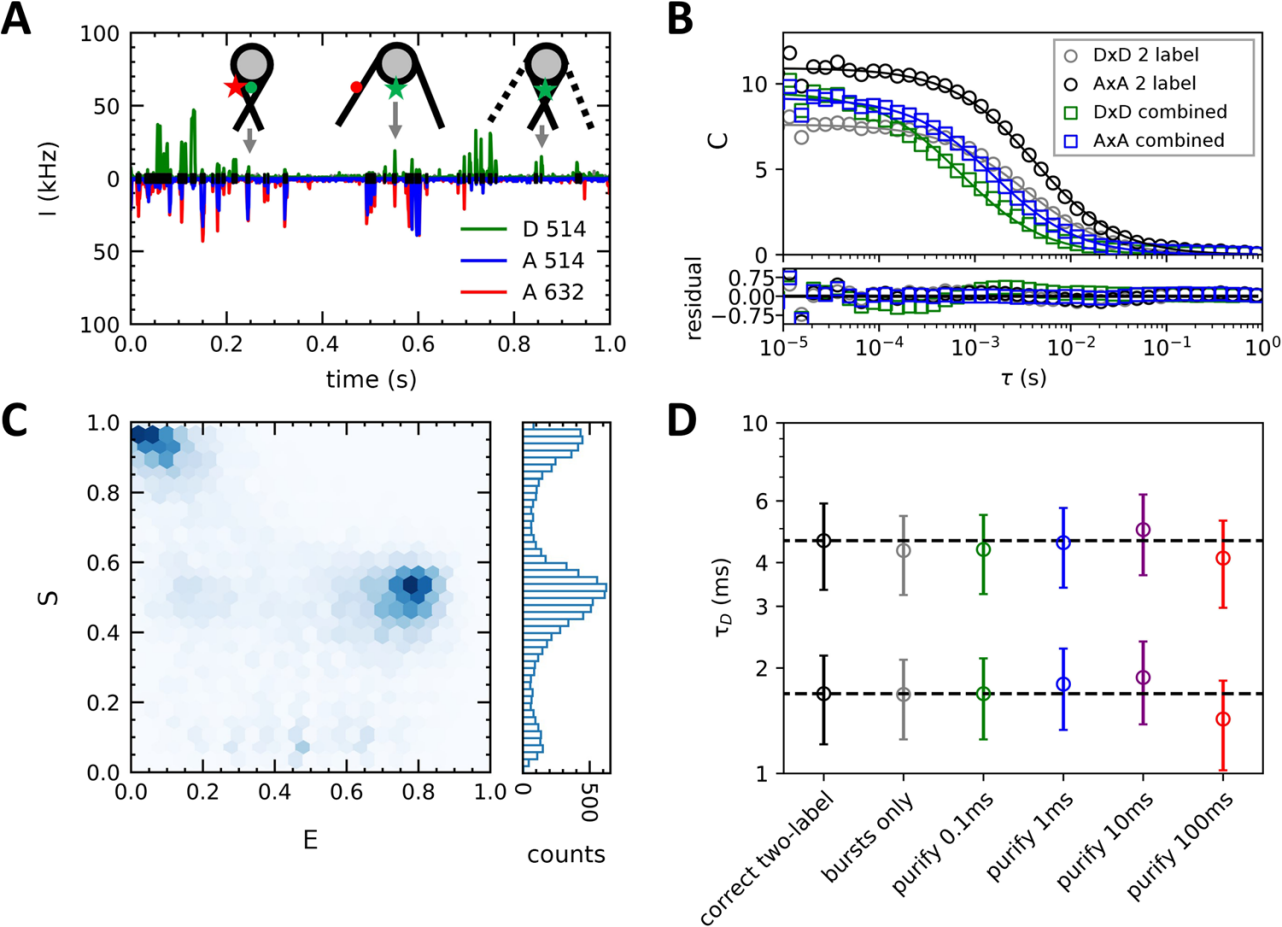
**Optimizing the purified time allows for better retrieval of diffusion times.** **A)** Typical time trace showing bursts from low and high FRET populations, as well as donor and acceptor-only populations. FRET emission is shown in blue. Donor emission is shown in green. Acceptor emission under acceptor excitation is shown in red. Detected bursts are highlighted in black at $I = 0$. **B)** The correlation function of acceptor and donor emission of all photons (’combined’, blue and green squares) and only photons from the two-labeled population (’2 label’, black and grey circles). **C)** E, S histogram of detected bursts shows the largest population at $S = 0.5$, with bleached populations at $S = 1$ and $S = 0$. **D)** Fitted diffusion times for 30 nm (top) and 10 nm (bottom) particles show minor deviations, with $t_{purify} = 1$ ms as optimal in the first case. The dotted line represents the original diffusion time of the two-labeled species

For mobile particles, diffusion makes fitting the timescale of conformational dynamics more unreliable, as shown in Fig. S2. In addition, since only short bursts of individual particles are recorded, alternative methods for determining transition rates such as hidden Markov model analysis [51] that could be applied for the experiments on immobilized particles could not be applied here either. Figure S2E shows that kinetic rates for mobile particles can therefore not be determined as accurately as for the immobile particles in Fig. 2. Nevertheless, independently determined values of each conformational state’s FRET value, for example by using immobilized particles, may help to determine transition rates through correlation analysis, since this would reduce the number of free parameters in the analysis.

### Combining Burst Selection and Purified FCS for Correlation Analysis

In real experiments, there are additional complications that may further affect the accuracy of a FRET-FCS experiment. In addition to overlapping timescales, bleaching of one of the fluorophores can have a significant impact on single-pair FRET measurements. To simulate this, we included 20 % of donor-only and 20 % of acceptor-only molecules. We added a fourth population of considerably smaller size ($R = 1$ nm) with the same concentration and spectral properties as the other donor-only species to represent impurities in the measurement buffer. Moreover, spectral leakage is hard to avoid in experiments, and we set $\alpha = 0.1$.

A typical time trace of such a simulation is shown in Fig. 3A. Detected bursts were highlighted as black bars at $I=0$. Such bursts originate from high and low FRET states and molecules with one of the labels bleached, for which it is impossible to determine their conformation. The combination of these additions had a considerable effect on the obtained correlation function. Figure 3B shows the comparison between the FRET and non-FRET auto-correlation functions of the entire measurement (blue and green) and that of the selected two-labeled bursts (black and grey). Not only are the amplitudes different, which would cause *K* to be incorrectly calculated, but the decay time also differed, which affects the extracted kinetic and diffusion time constants. Mono-exponential fits to the correlation curves featured a large residual, indicating a profound influence of the introduced effects and complicating quantitative data analysis.

**Fig. 4:**
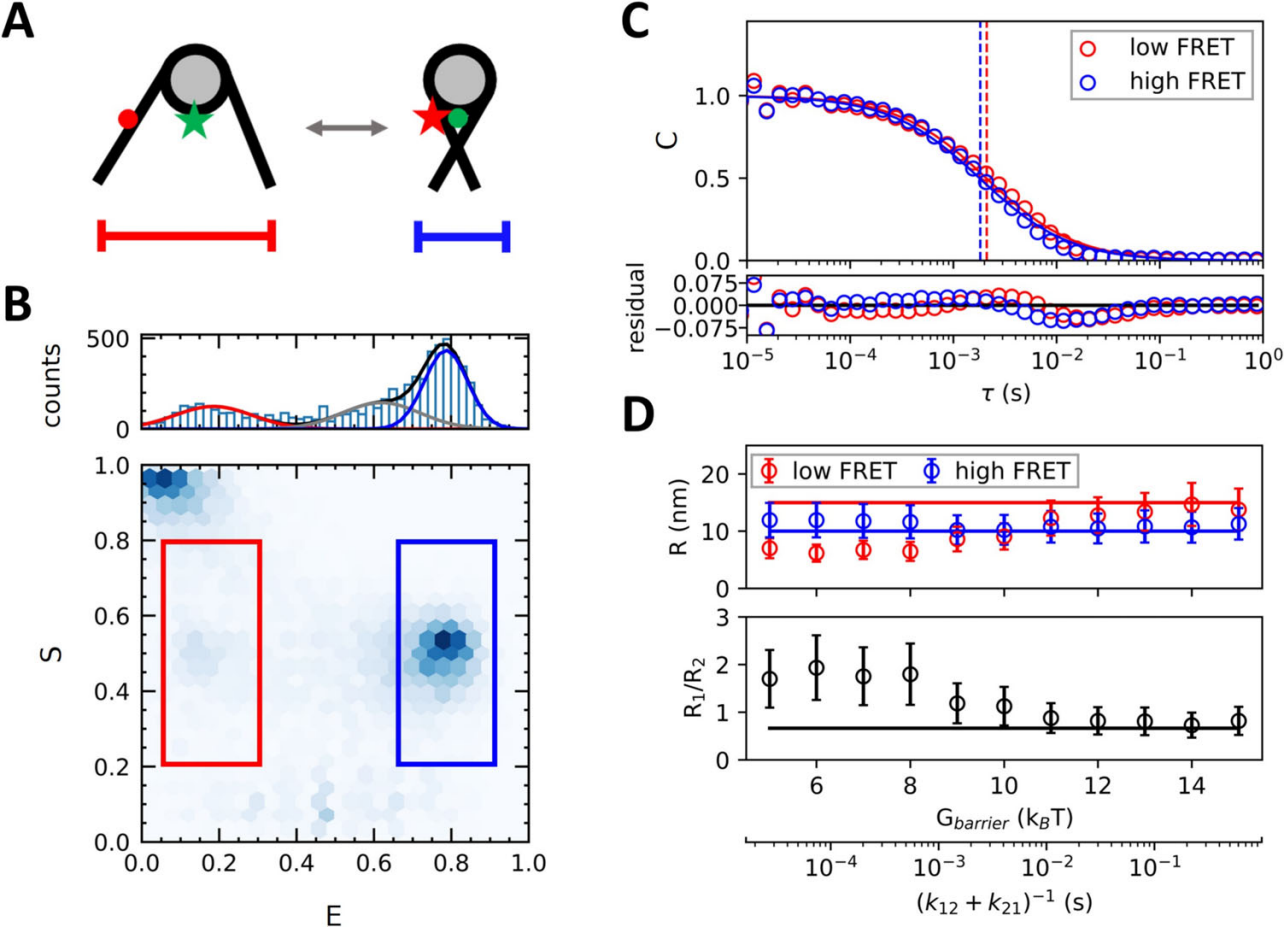
**Diffusive properties of FRET sub-populations can only be determined when kinetic relaxation times exceed the diffusion times.** Determining $R_{1} = 10$ nm and $R_{2} = 15$ nm through correlation analysis was possible with a kinetic relaxation time of more than 30 ms ($G_{barrier} > 11\, k_{B}T$). **A)** DNA unwrapping from nucleosomes yields larger conformation and lower FRET values. **B)** The E, S histogram of the detected bursts shows three main populations for double-labeled species ($S \approx 0.5$): low FRET (red), intermediate FRET (grey), and high FRET (blue) for $G_{barrier} = 12\, k_{B}T$ and $K = 4.5$. **C)** Purified correlation function of high FRET $E_{1} = [0.68,0.92]$ (blue) and low FRET $E_{2} = [0.05,0.30]$ (red) shows a clear difference in diffusion time between populations. $R_{1} = 10$ nm, $R_{2} = 15$ nm and $G_{barrier} = 12 \, k_{B}T$. **D)** Fitted sizes (top), as well as their ratio (bottom) for increasing $G_{barrier}$ show that these can only accurately be determined for $G_{barrier} > 11\, k_{B}T$. The high FRET state with $R_{1} = 10$ nm is shown in blue and the low FRET with $R_{2} = 15$ nm is shown in red

To alleviate these problems, we combined burst selection with purified FCS. First introduced by Laurence et al. [52], purified FCS aims to minimize contributions of fluorescence originating from single-labeled species, specific FRET subpopulations or fluorescent contaminations to the correlation function. It does so by analyzing photons originating from selected bursts and from time windows $t_{purify}$ before and after them. An in-depth explanation of this technique can be found in the methods section. To implement this, we first performed burst analysis on the time trace and determining stoichiometries and FRET values of all bursts, shown in an *E*, *S* histogram in Fig. 3C. As expected, we observe four distinct populations. At $S = 1.0$, $E = 0.0$ there is the donor-only population. $S = 0.0$ there is the acceptor-only population. The double-labeled population at $S = 0.5$, features two FRET sub-populations at $E = 0.8$ and $E = 0.1$. Next, we applied purified FCS to the $S = 0.5$ population, as visualized in Fig. S3A, and constructed their correlation functions, as shown in Fig. 3B.

The optimal purified time $t_{purify}$ likely depends on the diffusion time of the particle. To test this we calculated the correlation functions of two different sets of simulations with $R = 10$ nm and $R = 30$ nm, shown in Fig. 3D. Expected diffusion times from Eq. 24 were 1.6 ms and 4.8 ms. Fitted values of the double-labeled correlation were $1.7 \pm 0.5$ ms and $4.6 \pm 1.3$ ms respectively. The burst-only correlation (grey) shows a deviation from the desired diffusion time for R = 30 nm. This is a consequence of selecting only segments of a particle’s movement through the excitation beam that have sufficiently high intensity, which reduces the burst lengths. When we compare this to purified FCS, we found that $t_{purify} = 1$ ms gave us the best fit: calculated diffusion times were $1.8 \pm 0.5$ ms and $4.4 \pm 1.1$ ms. The fitted diffusion time for $t_{purify} = 100$ ms provides us with further insight into the limits of this method. As $t_{purify}$ increases, more fluorescence from undesired particles such as the $R = 1$ nm donor-only population representing fluorescent pollution, will be included in the analysis. The choice of $t_{purify}$ is a trade-off: too short clips the burst-photon only signal, and too long will include fluorescence from other sources. Despite this, the results were within the error of fit, showing relatively minor deviations from the initial value. While for most analyses these differences will not significantly alter the outcome, optimizing $t_{purify}$ can be important when distinguishing small changes in diffusive properties between conformational states.

### Sub-Population Analysis of Translational Diffusion

In the previous section, we were able to distinguish different fluorescent populations through a combined burst analysis and purified FCS approach. Here we extract the size of different conformations, using nucleosomes as a representative model. DNA unwrapping from nucleosomes increases the radius compared to closed nucleosomes [48] as the unwrapped DNA extends out from the otherwise disc-like nucleosome core particle. Unwrapping also leads to a lower FRET value, as shown in Fig. 4A. The implementation of this model in our simulations is shown in Fig. S4.

For values of $\Delta G= 1.5\, k_{B}T$, $G_{barrier}= 12\, k_{B}T$ (corresponding to $K = 4.5$, $k_{12}=6\,s^{-1}$ and $k_{21}=27\,s^{-1}$, so much slower than diffusion), and conformational state-dependent particle sizes $R_{1} = 10$ nm and $R_{2} = 15$ nm, we found three separate FRET populations in the *E*, *S* histogram when selecting for $0.2< S < 0.8$. Figure 4B shows these low, intermediate, and high FRET populations obtained through burst analysis. After applying a purified time of 1 ms, we fitted Eq. 23 to the auto-correlation curves of both the low- and high FRET populations in Fig. 4C. By splitting the FRET values into separate sub-populations and removing the intermediate FRET populations, the effects of conformational dynamics within bursts are minimized. The two correlation curves are slightly offset and the fitted values of the diffusion time for $G_{barrier}= 12\, k_{B}T$ were $1.8 \pm 0.5$ ms and $2.1 \pm 0.5$ ms for the smaller and the larger particles, separating the diffusion time of the two species based on their FRET efficiency.

Accurate application of this method requires sufficiently slow kinetic rates though, as is evident from Fig. 4D. Here we plotted the input radii of state 1 and state 2 against their fitted values, as well as the ratio between these two radii. For $G_{barrier} > 11 \, k_{B}T$ the input radii could be extracted reliably. Interestingly, for $G_{barrier} < 11 \, k_{B}T$ we see a consistent underestimation of the low FRET population’s particle size, even to the extent where this population’s radius is estimated to be smaller than that of the high FRET population. This is due to an intrinsic sampling bias towards shorter bursts for the low FRET population when transitions are much quicker than the diffusion time. For instance, $G_{barrier} = 8 \, k_{B}T$ corresponds to a lifetime of the low FRET state $\tau _{2}= 0.66$ ms, less than half the particle’s diffusion time. As a consequence, most measured bursts will include at least one transition, shifting their calculated *E* value upwards. The bursts that do not contain transitions are then more likely shorter bursts, resulting in a smaller calculated particle size. The ability to resolve meaningful differences in particle size between FRET sub-populations is therefore dependent on transitions occurring at a rate slower than that species’ typical diffusion time.

**Fig. 5:**
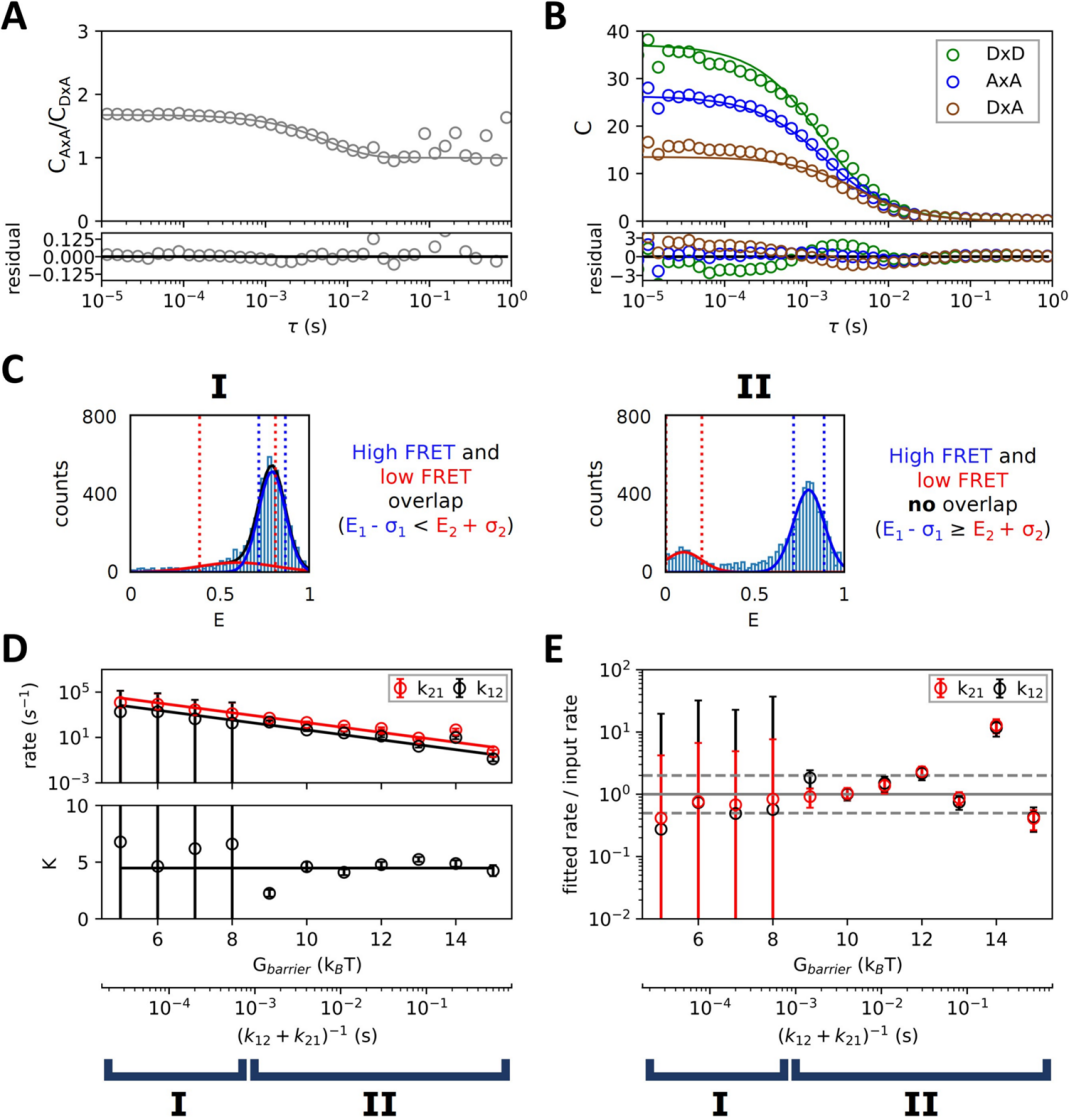
**Conformation state lifetimes between 100 µs and 100 ms can be reliably determined using a combined burst analysis - purified FCS approach.** **A)** Dividing the FRET auto-correlation by the FRET$\times$non-FRET cross-correlation divides out most contributions from diffusion. Conformational dynamics can then be determined from the remaining function. $G_{barrier} = 10\, k_{B}T$. **B)** Multi-correlation fit of non-FRET auto-correlation ($D\times D$, green), FRET auto-correlation ($A\times A$, blue), and non-FRET$\times$FRET cross-correlation ($D\times A$, brown). Differences in correlation function shape are a result of conformational dynamics. $G_{barrier} = 10\, k_{B}T$. **C)** Overlap of FRET populations was determined by the fitting histogram of the bursts’ FRET values. Case I: transitions occur at the same timescale as, or faster than, diffusion. This causes the FRET subpopulations to merge in the histogram. Case II: transitions occur slower than diffusion, so FRET subpopulations are separate. Constraints are imposed on the fit of the correlation function depending on the case. **D)** Transition rates $k_{12}$ and $k_{21}$ (top), and equilibrium constants *K* (bottom), fitted with method from A) for increasing $G_{barrier}$ and $R_{1} = 10$ nm, $R_{2} = 15$ nm. Horizontal lines are the input value. Fit constraints were determined by FRET population overlap (case I and II). **E)** Ratios of the fitted transition rates to their input rates from C) show slight underestimation for short lifetimes and increasing deviation at long lifetimes. Dashed lines show a factor-2 difference. $\Delta G=1.5\, k_{B}T$ for all figures

### Extracting Conformational Dynamics with Purified FCS

After having determined the limits of discerning the mobility of different FRET populations, we used the simulations to define a range of measurement parameters within which the conformational dynamics can be captured. Purified FCS with $t_{purify} = 1$ ms was applied to all bursts with $0.2< S < 0.8$. We compare two methods for the analysis of mixed population FRET-FCS and applied corrections to account for differences in conformation-dependent particle size.

To isolate conformational dynamics and to cancel the contribution of diffusion in the correlation curve, Torres et al. [17] divided the FRET auto-correlation function $C_{A \times A}$ by the FRET $\times$ non-FRET cross-correlation function $C_{D \times A}$. Under the assumption that the diffusion coefficient of both states is identical, the diffusion term cancels, and the resulting ratio of correlation functions can then be fitted to $C_{A \times A}/C_{D \times A}$ as described in Eqs. 27 and 28. While the two measurement channels provide more options to choose correlation functions as numerator or denominator, the FRET auto-correlation $C_{A \times A}$ typically has the largest amplitude increase due to conformation dynamics. Conversely, the cross-correlation $C_{D \times A}$ has the largest decrease in amplitude from contributions of conformational changes. The division of these correlation curves then results in the largest contrast.

Figure 5A shows this method applied to simulations with particle sizes $R_{1} = 10$ nm and $R_{2} = 15$ nm. $G_{barrier} = 11 \, k_{B}T$ yields conformational state lifetimes $\tau _{1} = 59.9$ ms and $\tau _{2} = 13.4$ ms. From the simulations however, we fitted $\tau _{1} = 36.9 \pm 10.2$ ms and $\tau _{2} = 9.9 \pm 2.4$ ms. At the several milliseconds, we observed a transition attributed to conformational changes, from a $C_{A\times A}/C_{D\times A}$ ratio $\sim$ 1.7, to the larger timescales, where the ratio becomes 1.

A second method, proposed by Price et al. [18], applies global fits to $C_{A \times A}$, $C_{D \times D}$, and $C_{D \times A}$ instead. Figure 5B shows an example of such a global fit to the same simulation data as in A. In contrast to Price et al. [18], we did not assume that the three correlations share the same $\tau _{D}$. In the case of $C_{D \times D}$ there is a significant deviation from a mono-exponential decay. This results in systematic deviations of the residual for all three correlations, as they share the same incorrectly fitted *K*. it is likely that trace amounts of the much smaller D-only contaminating species were included in the purified FCS selection. The fitted lifetimes were $\tau _{1} = 10.3 \pm 5.2$ ms and $\tau _{2} = 4.0 \pm 2.0$ ms.

When the kinetic relaxation time is shorter than the diffusion times (Fig. 5C, case **I**), transitions are more likely to occur within a burst. This prevents us from distinguishing discrete populations by burst FRET value alone. In this case, we took $R_{1}$ = $R_{2}$, as the results in the previous section show that in this situation, differences in particle sizes could not be accurately determined. We also constrained $E_{1}$, $E_{2}$ to $f_{1}E_{1}+f_{2}E_{2}=E_{overlap}$, with $f_{1}$ the fraction of molecules in state 1, $K=f_{1}/f_{2}$ the equilibrium constant and $E_{overlap}$ the fitted FRET value of the overlapping FRET population.

When the kinetic relaxation time exceeds diffusion times (Fig. 5C, case **II**), we can reliably determine values for $E_{1}$, $E_{2}$, $R_{1}$, and $R_{2}$ from burst analysis. Since conformational changes affect the size of the particle in our simulations, corrections to the fitted correlation function from differences in diffusion time were applied according to Eqs. 32-34.

We applied both methods for extracting lifetimes to simulations with increasing $G_{barrier}$. For each measurement, we used one of two different sets of constraints on the fitted parameters. We selected between them by comparing the FRET value *E* and width $\sigma$ of the fitted FRET histogram populations. If these populations would overlap ($E_{1} - \sigma _{1} < E_{2} + \sigma _{2}$), case **I** was applied. If not, case **II**.

For particles with $R_{1}=10$ nm and $R_{2}=15$ nm, representing a single nucleosome, the method proposed by Torres et al. [17] yielded the most reliable results. This is shown in Fig. 5D. Here we fitted transition rates $k_{12}$ and $k_{21}$ (top) and equilibrium constants *K* (bottom) for increasing $G_{barrier}$. The ratio between the fitted and the input values of $k_{12}$ and $k_{21}$ are also shown in Fig. 5E. For $G_{barrier} < 9\, k_{B}T$, corresponding to lifetimes of less than $\tau _{1} = 8.1$ ms and $\tau _{2} = 1.8$ ms, $k_{12}$ and $k_{21}$ yielded large standard errors and the equilibrium constant *K* could not be determined very accurately. For larger lifetimes of $G_{barrier} > 12\, k_{B}T$, the fitted equilibrium constant deviated little from the expected value, although the separate reaction rates could be off by an order of magnitude. This is not surprising, as at these timescales the correlation function will have almost completely decayed by diffusion.

An extensive comparison between methods for $R_{1}=10$ nm, $R_{2}=15$ nm (representing breathing of a nucleosome), and $R_{1}=30$ nm, $R_{2}=90$ nm (representing the compaction and decompaction of a nucleosome array [49]) simulations can be found in Figs. S5 and S6. We also included a variation of the second method, where we only fit the $C_{D \times D}$ and $C_{D \times A}$ channels. However, both variations showed similar results to fitting $C_{A\times A}/C_{D\times A}$.

It is clear from Fig. 5A and B why conformational changes at large $\tau$ are so hard to quantify with either of the methods: as diffusion causes correlation functions to decay to zero, the influence of noise from background fluorescence, bleaching, and artifacts from purified FCS on small amplitudes become more pronounced. Dividing small correlation amplitudes at $\tau > 0.1$ s increases the noise, as can be seen in Fig. 5A.

**Fig. 6:**
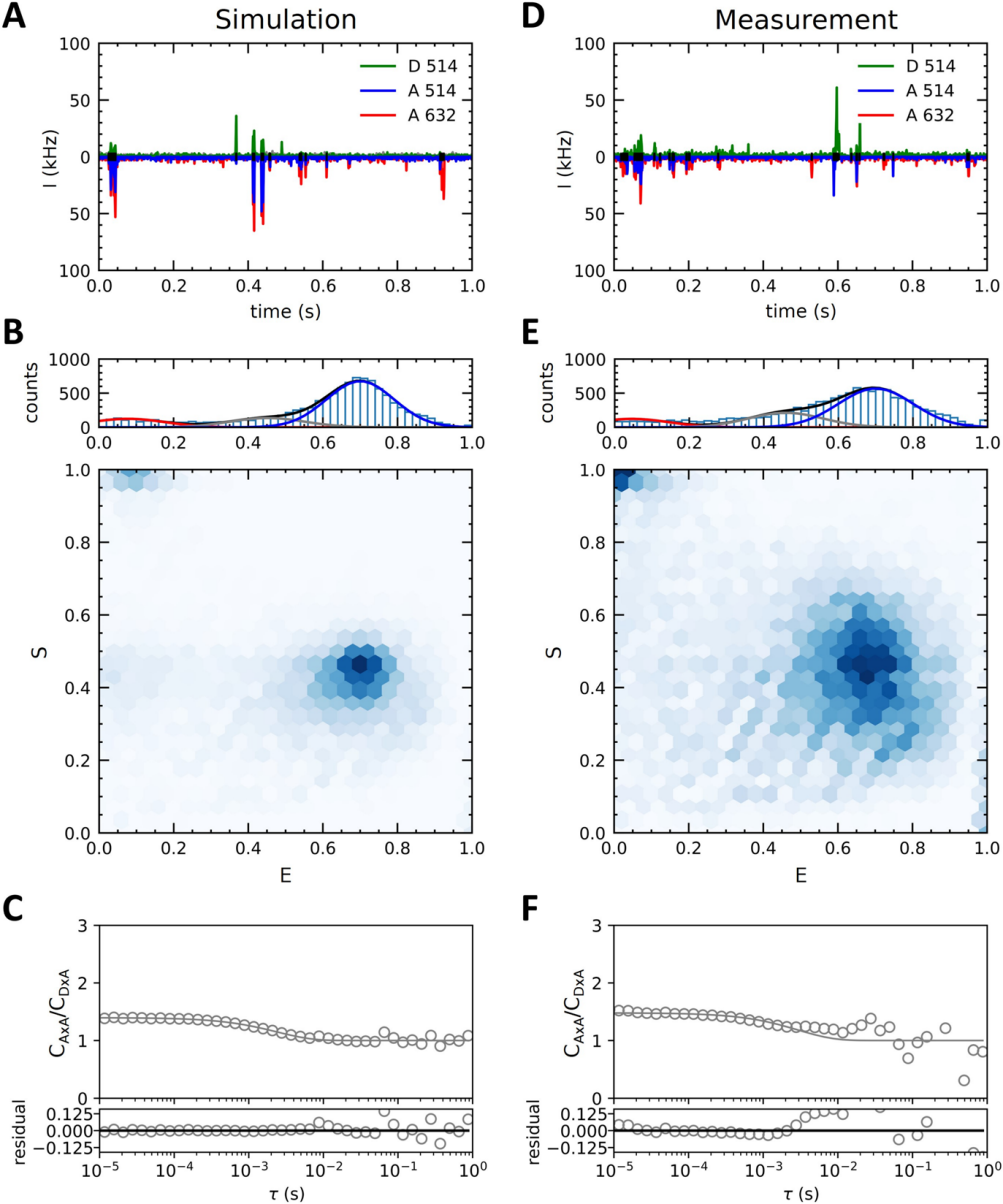
**Comparison of simulated (left column) and experimentally measured (right column) spFRET-labeled nucleosomes.** The two primary FRET burst populations are correctly quantified, however some intermediate sub-states of nucleosome breathing dynamics can not be captured fully. **A)** Time trace of simulated nucleosomes shows the presence of low FRET, high FRET and single labeled populations. To avoid overlap, we displayed the signal of the red detector as negative intensities. **B)** E, S histogram of detected bursts of simulated nucleosomes shows a majority of the high-FRET population. **C)** Division of the FRET auto-correlation by the FRET$\times$non-FRET cross-correlation of the simulated nucleosomes divides out most contributions from diffusion. $\tau _{1, sim} = 17.6 \pm 0.6$ ms and $\tau _{2, sim} = 3.37 \pm 0.10$ ms. **D)** Time trace of measured FRET-labeled nucleosomes. **E)** E, S histogram of detected bursts of nucleosomes shows larger variance in E, S, of burst populations. The fraction of intermediate populations is larger than in the simulation. **F)** Division of the FRET auto-correlation by the FRET$\times$non-FRET cross-correlation of the measured nucleosomes yields an initial decay of the curve attributed to conformational dynamics, but the presence of intermediates with longer lifetimes are not captured in the fit. $\tau _{1, \exp } = 20.3 \pm 2.2$ ms and $\tau _{2, \exp } = 4.4 \pm 0.4$ ms

Nevertheless, conformation dynamics can be recovered up to lifetimes larger than the diffusion time. The improvements from combining purified FCS with ALEX selection and subsequent analysis of the correlation functions described in this section are considerable. For rapid transitions with lifetimes shorter than the diffusion time, values for $E_{1}, E_{2}$ and *K* are hard to disentangle, but the changes in *E* are temporally separated from diffusion and can be fitted from the correlation curve. For slow transitions with lifetimes longer than the diffusion time, one can fix FRET levels and separate diffusion times from the different FRET populations, which leaves the transition rates $k_{12}$ and $k_{21}$ as the only free fitting parameters for the correlation curves. These values, however, become more inaccurate as they go beyond the correlation function’s diffusion decay time. By combining both, lifetimes between 100 µs to 100 ms can be extracted with shot-noise limited accuracy from the correlation functions of freely diffusing particles even when conformational states differ in size.

### Experimental Validation of Conformational Dynamics Analysis Applied to FRET-Labeled Nucleosomes

To verify the applicability of our method to the analysis of spFRET experiments, we performed measurements on FRET-labeled nucleosomes. Experimental values were $\tau _{1, \exp } = 20.3$ ms and $\tau _{2, \exp } = 4.4$ ms, corresponding to $G_{barrier} = 9.92\, k_{B}T$ and $\Delta G = 1.53\, k_{B}T$. The simulations show time traces (Fig. 6A) and burst populations (Fig. 6B) that reproduce the distinct low- and high-FRET populations with simulations done in the previous sections. The *E*, *S* histograms that result from burst analysis are also similar, though the experimental histogram features broader peaks and a larger fraction of donor-only nucleosomes. Apparently, we underestimated the various noise factors. However, increasing signal-to-noise by increasing the laser intensity would further increase the fraction of bleached fluorophores and can potentially alter the observed kinetics when a significant fraction of the bursts includes a bleaching event. Figure 6C shows the applied $C_{A \times A}/C_{D \times A}$ fit method, obtaining lifetimes of $\tau _{1, sim} = 17.6 \pm 0.6$ ms and $\tau _{2, sim} = 3.37 \pm 0.10$ ms, in line with the previous results in Fig. 5.

Although the measured time traces and burst populations of the nucleosomes in Fig. 6D and E show similar intensities and burst frequencies as those in the simulations, we measured a larger fraction of intermediate FRET bursts, as is evident from the grey population in the FRET histogram. This could indicate that our simple two-state model is insufficient to describe changes in nucleosome conformations. In addition, longer-lived intermediates appear in the $C_{A \times A}/C_{D \times A}$ curve in Fig. 6F, preventing the correlation curve ratio from decaying to 1 as per the model, and adding additional noise at longer $\tau$.

## Discussion and Conclusion

In this work, we investigated the limits of ALEX spFRET-FCS measurements and proposed methods for improving the accuracy of extracted kinetic parameters for conformational changes. To do so we simulated the entire experiment, including variations in diffusion, shot noise, impurities, optical properties of the microscope, time characteristics of the alternating excitation, and burst selection. We showed that determining conformational state lifetimes is limited by photo-bleaching, contaminations, and lack of knowledge of the particle’s FRET levels. In addition, we showed that variations in particle size due to conformational changes could be determined only when lifetimes were considerably longer than the timescale of diffusion. By performing a combined burst selection and purified FCS analysis we were able to accurately recover lifetimes between 100 µs and 100 ms. Corrections for conformation-dependent particle sizes were included to allow for the fitting of lifetimes several orders of magnitude longer than diffusion times.

One of the main limiting factors in determining conformational state lifetimes is the intensity of the measured fluorescence signal. For Fig. 2 we chose $I_{0}$ = 100 kHz, which is in the experimental range of both FCS [48] and TIRF [53] measurements. For typical TIRF experiments though, correlation analysis of sub-millisecond phenomena is not possible due to camera integration times that exceed milliseconds [54]. Similarly, the duration of single-molecule time traces of immobilized molecules is limited by the photo-bleaching of either of the FRET labels. Even for 60 s traces of immobilized molecules, the noise in the correlation function at $\tau<$ 10 $\upmu$s was considerable for both $G_{barrier}=3\, k_{B}T$ and $9\, k_{B}T$ curves. In addition, for lifetimes in the microsecond range ($G_{barrier}<3\, k_{B}T$), the average number of emitted photons will be less than 1 during the dwell time of such a short-lived state at this excitation intensity. At such low photon numbers, the shot noise will be overwhelming, making accurate quantification of lifetimes impossible without higher laser power, which in turn increases photo-bleaching and blinking. The short-lived dark states that cause blinking further complicate quantitative dynamic analysis.

When molecules freely diffuse in solution, an additional time constant enters the correlation curve. This poses a problem when timescales of conformational changes and diffusive motion overlap. For correlation analysis, this means that when the timescale of kinetics exceeds the diffusion timescale ($\tau _{K} \gg \tau _{D}$), the correlation function becomes the weighted sum of both conformations, and the transition kinetics will be lost.

The fit to the FRET auto-correlation is influenced by the co-dependence of three terms: the two FRET values and the equilibrium constant. Fitting all three variables as free parameters did not converge to a stable result. Thus more knowledge of the system must be obtained to constrain the fits so that timescales of diffusion and conformational changes can be disentangled.

The three unknown parameters can, however, be determined before fitting the correlation curve through burst analysis. The use of ALEX has the added advantage of being able to discard bleached or otherwise single-colour populations. Bleaching during the trajectory of the molecule through the laser focus would not be excluded, and the stoichiometry and FRET value of such burst would be affected by the bleaching event. If bleaching rates exceed the diffusion time, this may be confused for a conformational switch if the selection criteria are not strict enough. For typical excitation intensities, this is not the case, even in the absence of oxygen scavenging systems [55]. Contributions from photo-physical effects at timescales shorter than that of diffusion, like blinking, can also introduce inaccuracies in determining the FRET and stoichiometry values of bursts. Blinking occurs most often from inter-system crossing into triplet states, with triplet state lifetimes in the microsecond timescale [39, 56]. Triplet states also have contributions in the correlation function, but using the equations introduced by Price et al. [18] their contributions can be isolated by making use of the fact that donor and acceptor triplet states are not correlated. In addition, burst selection algorithms like dual-channel-burst-search (DCBS) [57] have been developed to independently examine donor and acceptor photon streams, effectively limiting artifacts caused by blinking and bleaching. Experimentally the contribution of triplet states can be minimized by the addition of triplet state quenchers such as Trolox [58–60].

We used burst selection to purify the fluorescence signal from background fluorescence before correlation analysis. This method, however, introduces artifacts to the correlation function that could not be accounted for in the fit, depending on the choice of $t_{purify}$. Laurence et al. [52] used a longer time ($t_{purify}$ = 100 ms) than the one used here ($t_{purify}$ = 1 ms), but this is not the optimal choice for our measurements where a higher concentration of particles increases the chance of joining bursts from different molecules within $t_{purify}$. The optimal choice for $t_{purify}$, therefore, depends on the species’ diffusion time, concentration, and background signal. More generally, the criteria for burst identification are worth examining. Here we analyzed all photons within bursts, but kinetics can also be extracted by segmenting bursts according to the timescales of the studied kinetics, as described in time-resolved burst variance analysis (trBVA) by Terterov et al. [61]. In this case, the accuracy of extracted kinetic relaxation times close to the diffusion time is improved, at the expense of not being able to extract timescales an order of magnitude longer than the typical burst duration.

Diffusion coefficients of both states are often considered to be identical [17, 52], but this may not always be the case. Small differences in diffusion coefficient are particularly difficult to determine in FCS measurements, typically requiring at least a factor 1.6 difference [62], even with purified FCS. For more accurate determination of these values, several methods have been developed, such as dual-focus FCS [63], imaging FCS [64], and multi-scale FCCS [65]. Here, we calculated $\tau _{D}$ from a weighted average of the two subspecies, similar to the method implemented by Al-Soufi set al. [66]. Our analysis builds upon the seminal theoretical framework developed by Elson et al. [10] that does allow for $D_{1} \ne D_{2}$, but has not yet been developed for the FRET-dependent case. Pending such a more analytical approach, both methods of dividing the auto-correlation by the cross-correlation curves and globally fitting multiple correlations produced reliable results. Differences in diffusive properties and fluorophore brightness between molecules can also lead to biases in burst selection. Molecules with brighter or more numerous labels will be more likely to pass the intensity threshold set for burst selection, as will molecules that remain in focus for longer. These factors can however be corrected for [67].

The upper limit of conformational state lifetimes that we can extract from correlation analysis is defined by diffusion. While equilibrium constants in a two-state system could be accurately fit from the E, S-histogram, the values for the transition rates were not. When the equilibrium constant is obtained from the E, S-histogram, correlation analysis only needs one of the two transition rates $k_{12}$ and $k_{21}$. This is evident from the decay of $C_{conformation}$, which is dominated by the largest *k*, as the smaller *k* will typically be hidden by diffusion. Methods have been proposed to increase the range of accessible lifetimes, such as recurrence analysis of single particles (RASP) that uses FRET changes in consecutive bursts to measure longer than the typical diffusion time [68, 69]. In RASP, upon detecting a burst from a specific FRET subpopulation, FRET histograms of bursts occurring within a set recurrence interval are constructed. Variations in this histogram are then quantified according to the recurrence interval, as at shorter recurrence times the likelihood of consecutive burst originating from the same molecule is much higher than a different molecule entering focus. This allows for the probing of kinetics up to $\sim 100$ ms. Experimentally reducing diffusion by measuring in viscous media such as gels has also been applied [70, 71], although transition kinetics can be affected by this change in viscosity [72–74]. Alternatively, FCS on immobilized molecules can be used to extract lifetimes much longer than typical diffusion times, as long as bleaching allows [75, 76].

As an alternative to correlation analysis, probability distribution analysis (PDA) can calculate transition rates within bursts through analysis of the underlying photon distribution [77]. Since PDA analyzes deviations from a shot-noise-limited distribution of photons, it could offer advantages where the timescales of kinetics approach that of diffusion, similar to trBVA. For our current correlation-based approach, determining the boundaries between overlapping FRET states is based on the fitting of sub-populations. This introduces uncertainty in obtained values for *E* if there are multiple dynamic states present within one fitted population and could be avoided with PDA. In addition, PDA is less reliant on well-defined models. For instance, in the presence of more than two dynamically interchanging sub-populations, such methods can be expanded to any combination of static or dynamic populations [78–80]. In these cases, correlation analysis is further complicated by its prerequisite knowledge of fitting models and the number of conformational populations. Analyses based on maximum likelihood estimation (MLE) [81, 82] or more specific Hidden Markov models like H$^{2}$MM [83], which use photon arrival times to assign conformational states within bursts, can similarly be expanded to more than two states without prior knowledge of equilibrium constants or state-dependent FRET values.

Alternating excitation can obscure transitions of states with very short lifetimes. The modulation of the laser can lead to artifacts in the correlation function, visible as oscillations at timescales below 0.1 ms, whose amplitudes scale with the length of the excitation pulse. This makes the analysis of correlation functions from typical ALEX experiments where pulse periods are in tens of microseconds problematic [23]. Since these artifacts scale linearly with $C_{0}$, the division of two curves cancels their contributions, as shown in Fig. 5A. This is an advantage over the multi-correlation fit, although the fitted values of the current simulations did not differ significantly when $C_{D \times D}$ was excluded from the divided correlation fit. An additional term to the fit function that accounts for these modulation artifacts could also be introduced. Alternatively, two separate measurements could be performed. The first an spFRET burst-analysis experiment at $\sim$ 100 pM to obtain state-dependent FRET values, diffusion coefficients and bleaching fractions. The second an FCS experiment at $\sim$ 10 nM that could avoid the artifacts introduced to the correlation function by burst selection and ALEX modulation, while making use of the parameters obtained from the first experiment for its analysis. The applicability of this approach would, however, be contingent on the kinetics of the measured sample not being dependent on its concentration [84].

Using PIE, where the laser is modulated at (sub-) microseconds using pulsed lasers, as an alternative to ALEX also allows for fluorescent lifetimes to be extracted in addition to FRET and diffusion dynamics. However, the ns-pulses yield a low-duty cycle and limit measured fluorescence intensities. To compensate for this, excitation intensities can be higher than for continuous lasers and measurement times can be increased, until photo-bleaching kicks in. Fluorescence lifetime correlation spectroscopy (FLCS) [30, 33], however, has not been combined with two-colour excitation. It can be used with donor excitation only, as long as bleached populations are either prevented or discerned by fluorescence lifetime or FRET [85, 86]. Corrections in data analysis through dead-time corrections [87], improvements to photon detecting hardware [88], the use of nanophotonics [89, 90] and expanded experimental protocols [91] have also been proposed to expand the reliability and range of extracted parameters.

Comparing our simulations to experimentally measured nucleosomes illustrated the difficulty of reducing dynamic, three-dimensional structures to a single FRET value. Indeed, the presence of complex, multi-scale dynamics of nucleosome wrapping have also been shown both experimentally [92–95] and computationally [96–99]. Other biological systems might behave similarly when probed with FRET. In our simulation, we are capturing the shorter, millisecond scale process. Our simulation framework can be expanded to any number of intermediate conformational states by increasing the dimensions of the $G_{trans}$ matrix. It will be important to validate such complex analysis models with simulations like the ones we presented here to get a good grip on the effects of background signals, concentrations, sizes, and FRET levels on their outcomes.

In summary, we have simulated advanced ALEX spFRET-FCS experiments and tested the experimental boundaries for resolving accurate conformational state lifetimes, equilibrium constants, and FRET values of spFRET-labeled molecules. We have outlined and quantified the limits intrinsic to these measurements, originating from shot noise for short lifetimes and diffusion for long lifetimes. When optimized, spFRET-FCS in combination with ALEX is a powerful tool for analyzing conformational changes in molecules without requiring extensive prior knowledge of the system. Faster excitation schemes such as PIE are more suitable for probing lifetimes at the scale of microseconds. For longer lifetimes at the scale of seconds, constraints on diffusion or immobilization of the measured molecules are required.

## Supplementary Methods

Simulations were implemented using home-built python 3 software. The primary simulation code was used to simulate spFRET experiments, generating .npy files containing photon arrival times in the donor and acceptor channels. These were then converted to python data library dataframes with pandas, from which burst- and correlation fit data was extracted with the pycorrelate module and stored in .h5 format. Fitted parameters were exported as .csv file and plotted using a Python Matplotlib script.

Simulated experiments included the spectral and temporal settings of the microscope, as well as conformational dynamics of the nucleosomes. First, the conformational state of a set of molecules was calculated over time from a description of the energy landscape of the molecules. Each conformational state was assigned a diffusion coefficient *D* and FRET efficiency *E*. Using the diffusion coefficients, Brownian dynamics was used to simulate a 3D random walk for every molecule. The expected photon intensities were obtained by convolving the position of each molecule with a diffraction-limited focused laser spot in the shape of 3D Gaussian. The resulting intensity traces depend on a combination of the position, excitation wavelength, excitation intensity, excitation pattern and FRET value. Discrete photon arrival times were generated following a Poisson distribution of the expected photon intensity at any given timestep. This yields a time-tagged data file that has the same format as our experimental data. Finally, the obtained photon time tags were analyzed with a combined burst- and correlation analysis method.

### Conformational States

Given a two-state system with free energies $G_{1}$ and $G_{2}$, the transition probabilities between each state depend on the free energy matrix $G_{trans}$:

1 $$\begin{aligned} G_{trans} = \begin{pmatrix} G_{1} &  G_{1} + \Delta G + \Delta G^{\dag } \\ G_{2} + \Delta G^{\dag } &  G_{2} \end{pmatrix} \;, \end{aligned}$$

following Arrhenius kinetics [100, 101]:

2a $$\begin{aligned} k_{12}&= k_{s}e^{- (\Delta G + \Delta G^{\dag })/k_{B}T}\;,\end{aligned}$$

2b $$\begin{aligned} k_{21}&= k_{s}e^{- \Delta G^{\dag }/k_{B}T}\;, \end{aligned}$$

with $k_{12}$ the transition rate from state 1 to state 2. $k_{s}$ is a pre-exponential factor that is usually only weakly dependent on *T*. It can be interpreted as the number of transition attempts made per second by each molecule. In our simulations $k_{s}$ was fixed at $10^{6}$ s$^{-1}$. Conformational state lifetimes could then simply be calculated from each transition rate as $\tau _{1} = 1/k_{12}$ and $\tau _{2} = 1/k_{21}$. The equilibrium constant is defined as $K = k_{21}/k_{12} = N_{1}/N_{2}$, with $N_{1}$ the number of molecules in state 1. We then generated a transition rate matrix *k*:

3 $$\begin{aligned} k = \begin{pmatrix} -k_{12} &  k_{12}\\ k_{21} &  -k_{21} \end{pmatrix} \;. \end{aligned}$$

Finally, transition probabilities were determined by converting transition rates to probabilities, following Jones et al. [102]:

4 $$\begin{aligned} P = \begin{pmatrix} 1-P_{12} &  P_{12}\\ P_{21} &  1-P_{21} \end{pmatrix} \;, \end{aligned}$$

with elements

5a $$\begin{aligned} P_{12}(\delta t)&= k_{12}e^{-k_{12}\delta t} \;,\end{aligned}$$

5b $$\begin{aligned} P_{21}(\delta t)&= k_{21}e^{-k_{21}\delta t} \;, \end{aligned}$$

where $P_{12}$ is the probability for a molecule that starts in state 1 to end in state 2 within a given time $\delta t$. Note that this expression already takes into account the possibility of a molecule making multiple transitions within time step $\delta t$. Unless specified otherwise, the simulation time steps were taken to be 100 ns. Simulations with $G_{barrier} > 3\, k_{B}T$ were sped up by performing this state calculations with a 10 $\upmu$s time step. Initial states were assigned with a probability according to their transition rates: $P_{initial, \;1} = k_{21}/(k_{12}+k_{21})$, $P_{initial, \;2}= 1 - P_{initial, \;1}$.

### Brownian Dynamics

Simulations were performed on a fixed number of molecules. In simulations with mobile particles, we set *N* to five. This number was sufficient to include simultaneous entry of multiple molecules into the focus of the laser. The motion of each molecule was simulated by a 3D random walk, where the step size of its displacement was adjusted at each time step according to the diffusion coefficient *D* of the conformational state that in turn depends on its radius *R*, following the Stokes-Einstein equation at low Reynolds number:

6 $$\begin{aligned} D = k_{b}T/6\pi \eta R \;. \end{aligned}$$

The viscosity of the medium was taken to be that of water: $\eta = 1.0$ mPa$\cdot$s. The stepsize was then given by

7 $$\begin{aligned} L = \sqrt{2D\delta t} \;. \end{aligned}$$

Brownian motion yields a mean squared displacement (MSD) of

8 $$\begin{aligned} MSD(\tau ) = 2nD\tau = 6D\tau \;, \end{aligned}$$

with *n* the number of dimensions, being three, and time delay $\tau$. For a molecule that switches between two conformational states, the MSD becomes

9 $$\begin{aligned} MSD(\tau ) = 6(f_{1}D_{1}+f_{2}D_{2})\tau \;, \end{aligned}$$

with $f_{1}$, $f_{2}=1-f_{1}$ the fraction of time spent in state 1, 2, and $D_{1}$, $D_{2}$ their respective diffusion coefficients.

The concentration of the molecules was set by adjusting the volume of the simulation using periodic boundary conditions.

### Alternating Laser EXcitation (ALEX)

In our simulations we generated Alternating Laser EXcitation (ALEX) streams for 514 nm (donor) and 632 nm (acceptor) intensities, with a pulsed interleaved excitation pattern in which each laser was on for 400 ns, followed by a gap of 100 ns to avoid temporal overlap due to small lag-times of the laser response, as is the case in our experimental microscope. For 2-color excitation, this yields an ALEX period of 1 µs. This is shown in Fig. 1B.

### Fluorescence Intensities

For each molecule, we calculated the fluorescence intensity depending on its position, FRET state, and laser excitation wavelength. We simulated the laser beam as an extended 3D Gaussian with an axial asymmetry *a* along the propagation axis. The spot width of the laser as a function of the excitation wavelength $\lambda$ and the objective’s numerical aperture $N\!A$ was set by the diffraction limit:

10a $$\begin{aligned} \omega _{x}&= \omega _{y} = \omega _{xy}= \lambda /2N\!A \;,\end{aligned}$$

10b $$\begin{aligned} \omega _{z}&= a\omega _{xy} \;. \end{aligned}$$

Values of $N\!A=1.2$ and $a=8$ were chosen to match our experimental FCS setup. The effective focal volume was given by:

11 $$\begin{aligned} V_{eff} = \pi ^{3/2}\omega _{x}\omega _{y}\omega _{z} = \pi ^{3/2}\omega _{xy}^{3}a \end{aligned}$$

The fluorescence intensity of each molecule was proportional to the laser intensity at the position of the molecule, given by the following point spread function:

12 $$\begin{aligned} I = I_{0}\,\exp \left[ -\left( \frac{(x-x_{0})^2+(y-y_{0})^2+(z-z_{0})^2/a^2}{2\omega _{xy}^2}\right) \right] \;. \end{aligned}$$

Here $(x_{0},y_{0},z_{0})$ was the center of the beam and maximum intensity $I_{0}$. The pinhole of the confocal setup was included by discarding all fluorescence intensity originating from

13 $$\begin{aligned} \sqrt{(x-x_{0})^2+(y-y_{0})^2}<\frac{d_{pinhole}}{2M} \;, \end{aligned}$$

where the diameter of the pinhole $d_{pinhole}$ was 50 µm and the magnification of the microscope *M* was 60x.

Depending on the particle’s conformation, a fraction of the donor emission $f_{FRET}$ was converted to acceptor emission according to its FRET efficiency. In addition, a small fraction $f_{leakage}$ was transferred to the acceptor channel to account for spectral leakage. Background intensity $I_{bg}$ was added to match the dark currents of the APDs and residual light leaking into the microscope. Combining all these factors yields the total intensity:

$$\begin{aligned} I = I_{bg}+I_{0}{\sum _{i=1}^{N}}{\left\{ \begin{array}{ll} f_{exc} \;f_{FRET}\;f_{leakage}\; \exp \left[ -\left( \frac{x_{i}^2+y_{i}^2+z_{i}^2/a^2}{2\omega _{xy}^2}\right) \right] &  \;\;\sqrt{x_{i}^2+y_{i}^2}<\frac{d_{pinhole}}{2M}\\ 0 &  \;\;\text {otherwise.} \end{array}\right. } \;. \end{aligned}$$

Photons are emitted according to a Poisson process and follow the distribution

14 $$\begin{aligned} P_{n}(I, \delta t) = \frac{(I\delta t)^{n}e^{-I\delta t}}{n!}, \end{aligned}$$

where $P_{n}$ is the probability of emitting *n* photons within a timestep $\delta t$ for a given intensity *I*. For our simulations, as well as our experiments, the time step was $\delta t =$ 100 ns. Since $I \delta t \ll 1$, $P_{1} \gg P_{2, 3,...}$, and only one photon can be detected per time step, a time tag was generated at time step $\delta t$ if a randomly generated Poisson distributed number in the range [0, 1] is smaller than

15 $$\begin{aligned} P(I, \delta t) = I\delta t. \end{aligned}$$

Photon streams were split based on their excitation and emission spectrum wavelength: donor (*D*) - emitted by the donor fluorophore or acceptor (*A*) - emitted by the acceptor fluorophore, and 514 - excited by the donor wavelength or 632 - excited by the acceptor wavelength.

### Simulation Parameters

These parameters were used for the simulations unless specified otherwise:

- number of molecules: $N = 5$
- concentration: $$c = 50\cdot 10^{-9}\text { mol/m}^{3}$$
- laser excitation axial asymmetry: $a = 8$
- peak intensity 514 nm (Donor): $I_{0} = 150 \text { kHz}$
- peak intensity 632 nm (Acceptor): $I_{0} = 100 \text { kHz}$
- time step state transitions Eq. 5: $\delta t = 100 \text { ns}$ for $G_{barrier} \le 3\, k_{B}T$
- time step state transitions Eq. 5: $\delta t =$ 10 $\upmu$s for $G_{barrier} > 3\, k_{B}T$
- time step diffusion and photon emission Eq. 7: $\delta t = 100 \text { ns}$
- time step photon emission Eq. 14: $\delta t = 100 \text { ns}$
- Avogrado’s number: $$N_{a} = 6.022\cdot 10^{23}\text { mol}^{-1}$$

The following parameters are derived from these settings:

- simulation volume: $$V = N/(c\cdot N_{a}) = 1.66\cdot 10^{-16}\text { m}^{3}$$
- simulation box width: $r_{x} = r_{y} = r_{0}, r_{z} = a \cdot r_{0}, r_{0} = (V/a)^{1/3} = 2.75 \cdot 10^{-6}\text { m}$

The simulation can easily be extended to include misalignment of the two laser beams. Initial results showed that the effects were negligible for misalignments smaller than 0.5 $\mu m$. Another addition could be to increase the focus by under-filling the objective. This can be implemented by adjusting Eq. 10.

### Burst Analysis

The fluorescence intensity streams were analyzed using burst analysis [23]. Bursts were defined as a series of at least 25 consecutive photons with temporal gaps smaller than 200 µs. By exciting with both donor and acceptor wavelengths, the FRET value *E* and stoichiometry *S* for each burst was determined:

16 $$\begin{aligned} E = \frac{I^{A514}}{I^{A514}+I^{D514}} \;, \end{aligned}$$

17 $$\begin{aligned} S = \frac{I^{A514}+I^{D514}}{I^{A514}+I^{D514}+I^{A632}} \;. \end{aligned}$$

Here $I^{A514}$ is the number of photons emitted by the acceptor when excited at the donor excitation wavelength and $I^{D514}$ is the number of photons emitted by the donor when excited at the donor excitation wavelength. The above equations can be corrected for spectral leakage $\alpha$, differences in brightness between donor and acceptor fluorophores $\beta$, and direct excitation of the acceptor fluorophore $\delta$.

18 $$\begin{aligned} I^{A514}_{corrected} = I^{A514}-\alpha I^{D514} -\delta I^{A632} \;, \end{aligned}$$

19 $$\begin{aligned} E_{corrected} = \frac{I^{A514}_{corrected}}{I^{A514}_{corrected}+\beta I^{D514}} \;, \end{aligned}$$

20 $$\begin{aligned} S_{corrected} = \frac{I^{A514}_{corrected}+I^{D514}}{I^{A514}_{corrected}+I^{D514}+I^{A632}} \;. \end{aligned}$$

In our simulations we fixed $\beta = 1$, $\delta = 0$, similar to our experimental values. Spectral leakage $\alpha$ was varied between simulations. For our data analysis, these parameters were extracted from each simulation as described by Hohlbein et al. [23]. Populations were extracted by fitting the FRET histogram to three normal distributions, yielding the average FRET efficiency and its standard deviation.

### Correlation Analysis

Fluorescence Correlation Spectroscopy (FCS) allowed us to quantify diffusive motion that may vary with conformation through analysis of fluctuations in fluorescence intensity. The correlation function *C* is given as [103]:

21 $$\begin{aligned} C_{ij}(\tau )=\frac{\langle \delta I_{i}(t) \delta I_{j}(t+\tau )\rangle }{\langle I_{i}(t)\rangle \langle I_{j}(t)\rangle }=\frac{\langle I_{i}(t)I_{j}(t+\tau )\rangle }{\langle I_{i}(t)\rangle \langle I_{j}(t)\rangle } -1 \;. \end{aligned}$$

Here $\tau$ is the time between the arrival of a photon of color *i* and one of color *j*. $\delta I = I(t) - \langle I(t) \rangle$ is the deviation of the mean intensity. For $i = j$ we have an auto-correlation, otherwise we have a cross-correlation. The auto-correlation for a species undergoing diffusive motion is given by:

22 $$\begin{aligned} C(\tau ) = C_{0}\cdot C_{Diff}\;, \end{aligned}$$

23 $$\begin{aligned} C_{Diff}(\tau ) = (1+\frac{\tau }{\tau _{D}})^{-1}(1+a^{-2}\frac{\tau }{\tau _{D}})^{-1/2}\;. \end{aligned}$$

where *D* is the species’ diffusion coefficient and is defined by the diffusion time $\tau _{D}$ as

24 $$\begin{aligned} \tau _{D} = \frac{\omega _{xy}^{2}}{4D} \;. \end{aligned}$$

The amplitude of the correlation $C_{0}$ is the inverse of the number of particles in focus *N* and thus inversely proportional to the concentration *c*:

25 $$\begin{aligned} C_{0} = \frac{1}{N}=\frac{1}{V_{eff} \cdot c } \;. \end{aligned}$$

The objective of this work is to resolve conformational dynamics through changes in FRET efficiency with correlation analysis. A theoretical framework for FRET-value dependent correlation functions was introduced in Torres et al. [17]. Here we used an expanded formalism that includes contributions from spectral leakage $\alpha$ and differences in brightness between donor and acceptor fluorophores $\beta$, as described in Price et al. [18]. Unless specified otherwise, correlations were calculated from donor excitation: $C_{D514 \times D514}$ = $C_{D \times D}$

26 $$\begin{aligned} C_{D\times D}(\tau ) = \biggl [\;1\;+\;\frac{f_{1}f_{2}(E_{1}-E_{2})^{2}}{(1-f_{1}E_{1}-f_{2}E_{2})^{2}}e^{-\tau /\tau _{K}}\biggr ]\;, \end{aligned}$$

27 $$\begin{aligned} C_{A\times A}(\tau ) = \biggl [\;1\;+\;\frac{f_{1}f_{2}(E_{1}-E_{2})^{2}(\beta - \alpha )^{2}}{\bigl [\beta (f_{1}E_{1}+f_{2}E_{2})+\alpha (1-f_{1}E_{1}-f_{2}E_{2})\bigr ]^{2}}e^{-\tau /\tau _{K}}\biggr ]\;, \end{aligned}$$

28 $$\begin{aligned} C_{D\times A}(\tau ) = \biggl [\;1\;-\;\frac{f_{1}f_{2}(E_{1}-E_{2})^{2}(\beta - \alpha )}{\beta (1-f_{1}E_{1}-f_{2}E_{2})(f_{1}E_{1}+f_{2}E_{2})+\alpha (1-f_{1}E_{1}-f_{2}E_{2})^{2}}e^{-\tau /\tau _{K}}\biggr ]\;. \end{aligned}$$

$f_{i}$ is the fraction of molecules in state *i* and $E_{i}$ its FRET value for $i = 1, 2$, and $K = f_{1}/f_{2} = k_{21}/k_{12}$. $\tau _{K} = (k_{12}+k_{21})^{-1}$ is the relaxation time of the conversions between the two states of the molecule. Fluctuations in FRET from conformational changes increase the auto-correlation amplitudes of $C_{A\times A}$ and $C_{D\times D}$, while they decrease the cross-correlation amplitude of $C_{D\times A}$. The effects of both diffusive motion and conformational changes on the correlation functions are multiplicative under the assumption that all conformational states have identical diffusion coefficients:

29 $$\begin{aligned} C_{total} = C_{0} \cdot C_{Diff} \cdot C_{conformation} \end{aligned}$$

When conformational states have different diffusion coefficients, we used fluorescence intensity weighted diffusion components based on mixed species FCS [19, 20, 103, 104]. For *n* different species, the contributions from diffusion $C_{Diff,i}$ to the full correlation function depend on the fraction size $f_{i}$ of the population *N* and its relative brightness $Q_{i}$:

30 $$\begin{aligned} C_{i}(\tau ) = \frac{1}{N} \frac{{\sum _{i=1}^{n}f_{i}Q_{i}^{2}C_{Diff, i}(\tau )}}{\left( {\sum _{i=1}^{n}f_{i}Q_{i}}\right) ^{2}} \;. \end{aligned}$$

(Note that in Eq. 15 of [103] the brightness term in the numerator should be squared for a correct outcome). The cross-correlation is given by

31 $$\begin{aligned} C_{D\times A}(\tau ) = \frac{1}{N} \frac{{\sum _{i=1}^{n}f_{i}Q_{D,i}Q_{A,i}C_{Diff, i}(\tau )}}{\left( {\sum _{i=1}^{n}f_{i}Q_{D,i}}\right) \left( {\sum _{i=1}^{n}f_{i}Q_{A,i}}\right) } \;, \end{aligned}$$

with $Q_{D,i}$ the brightness of species *i* in the donor (D) emission channel and $Q_{A,i}$ the brightness of species *i* in the acceptor (A) emission channel. For a measurement of different FRET populations, each state’s fluorescence intensity *Q* is directly dependent on its FRET value *E*.

If one assumes all diffusion times to be equal, we obtain Eqs. 5 - 7 from [18]. Instead, we derived the complete correlation curve by averaging of subspecies’ diffusion components. Since the effect on the correlation function amplitude from changes in FRET state are already included in Eqs. 26 - 28, we neglected the denominator term of 30 and normalized to 1. The composite correlation functions for two species then become:

32 $$\begin{aligned} C_{Diff, \;D\times D}(\tau ) \!= \frac{f_{1}(1-E_{1})^{2}C_{Diff, 1}(\tau )+f_{2}(1-E_{2})^{2}C_{Diff, 2}(\tau )}{f_{1}(1-E_{1})^{2}+f_{2}(1-E_{2})^{2}} \;, \end{aligned}$$

33 $$\begin{aligned} C_{Diff, \;A\times A}(\tau ) = \frac{f_{1}E_{1}^{2}C_{Diff, 1}(\tau )+f_{2}E_{2}^{2}C_{Diff, 2}(\tau )}{f_{1}E_{1}^{2}+f_{2}E_{2}^{2}} \;, \end{aligned}$$

34 $$\begin{aligned} C_{Diff, \;D\times A}(\tau ) = \frac{f_{1}E_{1}(1-E_{1})C_{Diff, 1}(\tau )+f_{2}E_2(1-E_{2})C_{Diff, 2}(\tau )}{f_{1}E_{1}(1-E_{1})+f_{2}E_{2}(1-E_{2})} \;. \end{aligned}$$

Standard deviations $\sigma _C (\tau )$ of the correlation signal were calculated using bootstrapping as described in Wohland et al. [105]. Here, each measurement was split into equally long segments, and the standard deviation was calculated from the variations in those segments. Fits were weighted as $1/\sigma _C (\tau )$.

The autocorrelation measurement may also include a term accounting for molecules being excited to the triplet state, with a typical timescale of microseconds. Since this effect can be minimized by using appropriate triplet state quenchers, we did not include triplet state dynamics in our simulation.

Correlation functions are plotted with their fit residuals below. The scale of these residuals is ± 10$\%$ of the plot’s largest *C* amplitude.

### Combining Burst-Analysis with Purified FCS

To minimize contributions of fluorescence originating from single-labeled species, specific FRET subpopulations or fluorescent contaminations to the correlation function, Laurence et al. [52] introduced purified FCS, in which undesired segments of the photon streams were discarded before computing the correlation function. Here we built on this method and correlated only photons originating from specific *E* and *S* burst populations. As some of the criteria for burst selection might be too strict to fully characterize diffusion, we also included photons that arrived within time $t_{purify}$ before and after each of these bursts. By extending the burst selection with the purified time, we reduced the effect of clipping the signal by selecting only traces that have a high enough intensity to be identified as a burst [106].

### FRET Measurements on Nucleosomes

DNA substrates for FRET experiments on nucleosomes consisted of 185 bp containing a central Widom 601 sequence flanked by 19 bp linker DNA at both ends. The Cy3B and Atto-647N FRET labels were incorporated through PCR at the 7th and 84th base of the 601 sequence. When reconstituted, the DNA wraps around the histone core, bringing both labels together after one turn. This can be measured through changes in the FRET signal. The substrate sequence is available in the supplemental materials.

Nucleosomes were reconstituted through salt gradient dialysis, based on the method described in Kaczmarczyk et al. [107]. FRET-labeled 601 DNA was mixed with recombinant human histone octamers (EpiCypher 16-0001 : Histone Octamer, Recombinant Human) over a range of 0.8:1 to 1.6:1 histone:DNA ratios in a 2 M NaCl, pH 8.0, 1x TE buffer. Reconstitutions were performed with 400 ng 185 bp FRET-labeled 601 DNA and 200 ng 145 bp competitor DNA. Samples were pipetted into 10 kDa MWCO low-binding membrane cups (ThermoFisher 69572 : Slide-A-Lyzer MINI Dialysis Devices, 10K MWCO) and dialyzed from 2 M NaCl to $\sim$10 mM NaCl overnight. Optimal reconstitution samples were selected by gel-shift assay on 0.8 $\%$ agarose, 0.5 $\times$ TB gel, as shown in Fig. S7. Nucleosomes were diluted 1:500 in the measurement buffer to 100 pM. The measurement buffer was 128 mM NaCl, 0.01 $\%$ w/v BSA, 1 mM Trolox, 10 mM Tris-HCl pH 7.5. The measurement time was 10 minutes.

A home-built FCS set-up was used to perform the measurements. It consisted of an iChrome MLE multi-color laser, providing alternating excitation at 514 and 632 nm wavelengths. Excitation power was 0.6 mW and 0.3 mW, respectively. The excitation modulation pattern was the same as used in the simulations. The beam was focused into the sample with an Olympus 60x magnification, 1.2 NA, water immersion objective. Emitted light was focused through a 50 µm pinhole and then spectrally split between donor and acceptor emission wavelengths and detected by two Perking Almer SPCM-AQR-14 single-photon avalanche photodiodes. Photon arrival times were processed by a Picoquant TimeHarp 200 single-photon counting board.

Simulation parameters were based on these measurements. Intensities $I_{0}$ were determined by binning each burst over 0.1 ms (roughly one-tenth of the typical diffusion time) and taking the average of the ten largest burst intensities. FRET values were determined by fitting the FRET values of the burst population as described previously. Burst correction parameters $\alpha$, $\beta$, and $\sigma$ were extracted with the method described in [23]. Background intensities were found by fitting the histogram of the distribution of time between photon arrivals over 2 ms, attributed to sections without bursts, to Eq. 14 with $n = 1$. Particle concentrations, conformational state lifetimes, and state-dependent particle radii were determined through correlation analysis.

### Simulation Code Availability

The python code used to run the simulations can be found in a GitHub repository: https://github.com/JvN2/Simulating-smFRET-FCS-experiments.

## Supplementary Information

Below is the link to the electronic supplementary material.

Supplementary file 1 (pdf 4197 KB)

## Data Availability

Datasets are available on request.
