# Supplementary material for: Disentangling Timescales of Molecular Kinetics with spFRET using ALEX-FCS
Source: J Fluoresc. 2025 Feb 17;35(9):8551–70. doi: 10.1007/s10895-025-04187-0 (PMC12583438; doi:10.1007/s10895-025-04187-0)
Supplement: Supplementary file 1 — (pdf 4197 KB) [file 10895_2025_4187_MOESM1_ESM.pdf]

Disentangling timescales of molecular kinetics with spFRET using ALEX-FCS

Jeremy Ernst[1], Aditya Sane[2], and John van Noort[3, *]

[1] ORCID 0000-0001-5026-9890
[2] ORCID 0009-0000-9010-5321
[3] ORCID 0000-0002-9094-5598
[*]corresponding author, noort@physics.leidenuniv.nl, Biological and Soft Matter Physics, Huygens-Kamerlingh Onnes Laboratory, Leiden University, Niels Bohrweg 2, 2333 CA Leiden, The Netherlands

March 5, 2025

Supplementary Results

Simulating fluorescent signal from conformation state transitions

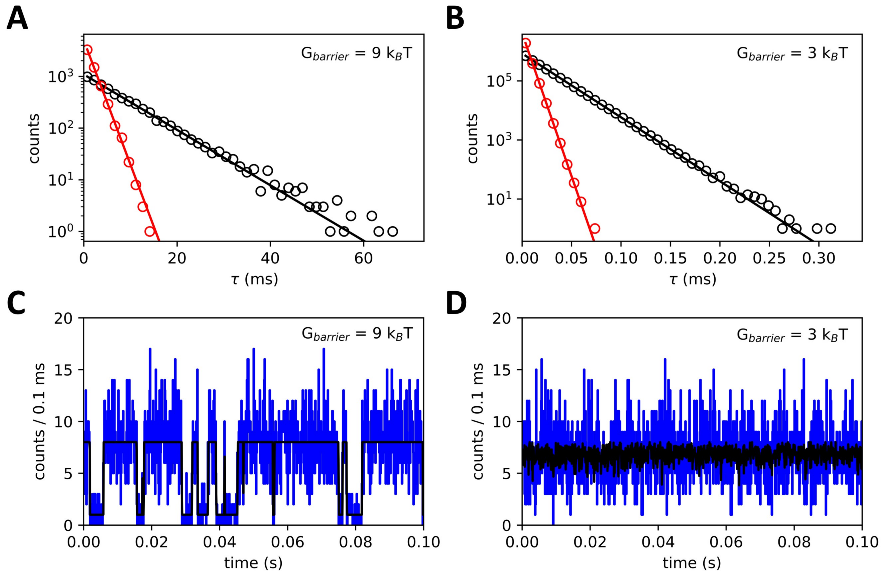

Figure S 1: **Comparison of lifetime distributions and time-traces of generated FRET states for $G_{barrier} = 9\,k_BT$ and $G_{barrier} = 3\,k_BT$ show harder to distinguish states for quick transitions A)** Distribution of lifetimes for $G_{barrier} = 9\,k_BT$ (corresponding to $k_{12} = 1.2 \cdot 10^2\ s^{-1}, k_{21} = 5.5 \cdot 10^2\ s^{-1}$). **B)** Distribution of lifetimes for $G_{barrier} = 3\,k_BT$ (corresponding to $k_{12} = 5.0 \cdot 10^4\ s^{-1}, k_{21} = 2.2 \cdot 10^5\ s^{-1}$). Black represents the lowest energy state, and red is the higher energy state. **C)**, **D)** Time trace of an immobile particle undergoing conformational dynamics with $G_{barrier} = 9\,k_BT$ and $G_{barrier} = 3\,k_BT$. The black line is the simulated intensity, blue graph shows the corresponding emitted photon distribution. The bin size is 0.1 ms. $\Delta G = 1.5\,k_BT$ for all figures.

Diffusion constraints on determining kinetic constants

We assessed the effect of diffusive motion on our ability to determine conformational state lifetimes accurately. Diffusive motion was simulated using a 3D random walk for five particles inside a box with periodic boundary conditions and a Gaussian excitation beam located in the middle of the box. Figure S 2 A visually represents this setup. Figure S 2 B shows the corresponding mean squared displacement (MSD) of all simulated particles, yielding a straight line, as is expected from a random walker. Figure S 2 C shows a representative time

trace of such an experiment, featuring bursts of photons when a single particle diffuses through the focus. Most bursts show a high FRET intensity, as is to be expected with $K = 4.5$ and $E_1 = 0.8$.

To determine the transition rates of particles with $G_{barrier} = 7\,k_BT$ and $\Delta G = 1.5\,k_BT$, we first correlated all photons during donor excitation (emitted from both donor and acceptor) and fitted eq. **??** to the obtained auto-correlation curve $C_{(D+A)\times(D+A)}$. Since both FRET and non-FRET photons are included, conformational changes do not affect the signal and we are left with only the diffusion contribution. This is shown by the grey curve in figure S 2 D. We then calculated the auto-correlation of the FRET photons $C_{A\times A}$. By taking eq. **??** and fixing the parameters of C_{Diff} to those found for the diffusion-only auto-correlation, we fitted eq. **??** to extract the conformation component. This is shown as the blue curve. When fitting, we fixed spectral leakage to zero and set the brightness of both fluorophores as equal i.e. $\alpha = 0$, $\beta = 1$, and left E_1, E_2, k_{12} and k_{21} as free parameters. The obtained rates are plotted in figure S 2 E. We observe that this method can not determine kinetic rates as accurately as for the immobile particles in figure **??**. While the trend of decreasing $G_{barrier}$ resulting in an increased fitted rate is apparent, neither the exact values of k_{12} and k_{21} nor their ratio K could be determined accurately.

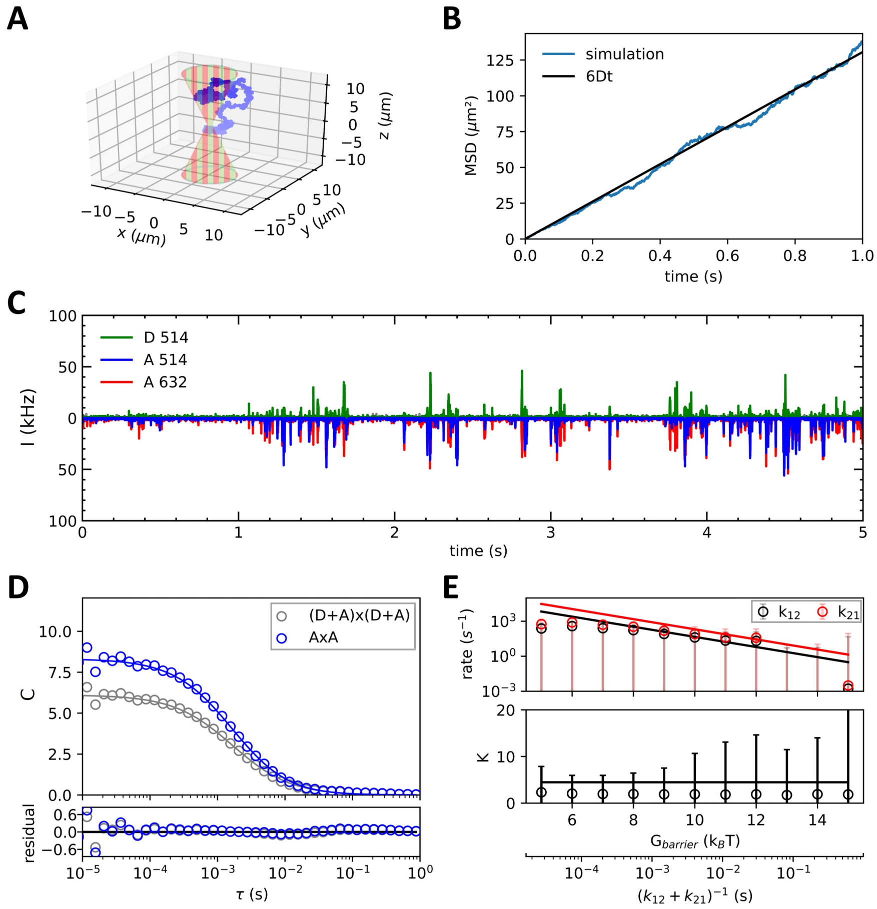

Figure S 2: **Diffusion limits the accuracy of transition rate measurement in single-pair FRET-FCS experiments. A)** Schematic representation of particles diffusing through the focus of alternating laser excitation. **B)** The mean squared displacement of diffusing particles. **C)** The time trace of a typical measurement shows fluorescence bursts originating from particles moving through the focus. Measured intensities are shown of donor emission under donor excitation (green), acceptor emission under donor excitation (FRET, blue), and acceptor emission under acceptor excitation (red). The bin size is 1 ms. **D)** Auto-correlation function of all photons (grey) fitted to diffusive motion, and FRET photons (blue) fitted to conformational dynamics with a fixed diffusion component corresponding to the all-photon correlation function. **E)** Fitted transition rates (top) and equilibrium constant K (bottom) for increasing $G_{barrier}$. Lines are the expected value from input. Fitted values show the predicted trend, but systematically underestimate K. $\Delta G = 1.5\, k_BT$ for all figures.

Combining burst selection and purified FCS for correlation analysis

Purified FCS was implemented by selecting all photons within the detected $S = 0.5$ bursts. The window of analysis was then expanded to include all photons a specific t_{purify} before and after each burst. Figure S 3 A shows the first 25 ms of figure **??** A with $t_{purify} = 10$ ms. Detected bursts of this population are indicated in black. Purified time around those bursts is shown in light grey. Time tags of bursts that were excluded from the purified signal, with $S < 0.2$ or $S > 0.8$ are shown in dark grey. The resulting correlation functions of all photons under donor excitation for different t_{purify} (blue, purple, and red), as well as the correlations of burst-only photons (grey), shown in figure S 3 B, almost overlap. Since we knew *a priori* which photons originated from the double-labeled populations, we also calculated the combined correlation function of both donor and acceptor time tags without impurities and bleached molecules (black). We adjusted the purified time parameter from 0.1 ms to 100 ms to obtain a good match with the input diffusion time. All correlation functions were normalized for a good visual comparison and appear highly similar. Still, minor differences between correlation functions exist. Single-exponential fits capture diffusion dynamics of the double-labeled species much better, with residuals within ± 0.01 for $\tau > 100\,\mu s$. Note that the deviations from the fitted curve at $\tau < 40\,\mu s$ are due to the alternating laser excitation. These oscillations are proportional to the correlation curve's amplitude and, therefore, overlap when the function is normalized.

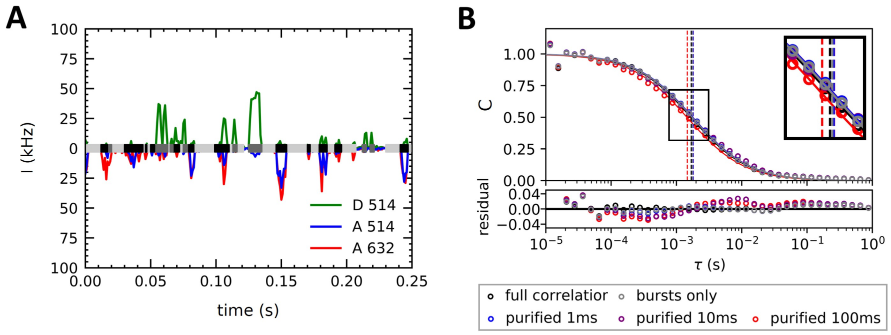

Figure S 3: **Purified FCS was implemented by expanding analyzed regions of the time-trace to before and after detected bursts. A)** purified time trace of the first 250 ms of panel A) selected for double-labeled bursts with $0.2 < S < 0.8$. Selected bursts are highlighted in black. An additional purified time of 10 ms is highlighted in light grey. Bursts of non-selected populations are highlighted in dark grey. The bin size is 1 ms. **B)** Correlation function of input double-labeled population (black), burst-only population (grey), and increasing purified time (1 ms - blue, 10 ms - purple, 100 ms - red. Range of residual is $\pm 5\%$.

Sub-population analysis of translational diffusion

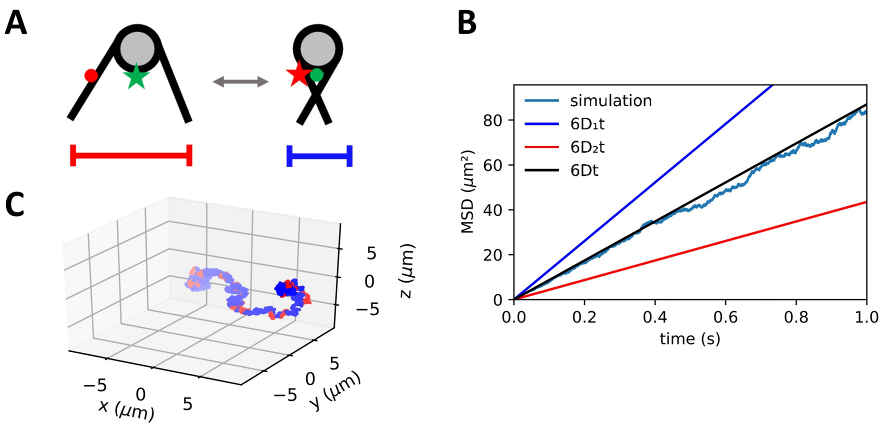

Figure S 4: **conformational states were assigned individual diffusion coefficients in the simulations. A)** DNA unwrapping from nucleosomes yields larger conformation and lower FRET values. We chose $\Delta G = 0$ for this example, meaning both states are equally likely. $K = 1$, $R_1 = 10$ nm (blue), and $R_2 = 30$ (red). **B)** The MSD of a two-state particle is proportional to the weighted average of the two diffusion coefficients (light blue line). The blue and red lines are the expected MSD for $R_1 = 10$ nm and $R_2 = 30$, and the black line is the weighted average. **C)** Changes in conformational state alter the particle's diffusion coefficient as apparent from the reduced extent of the red fractions of the trajectory. The particle is considerably more mobile in state 1 (blue) than in state 2 (red), as is to be expected from the difference in radius (eq. **??**).

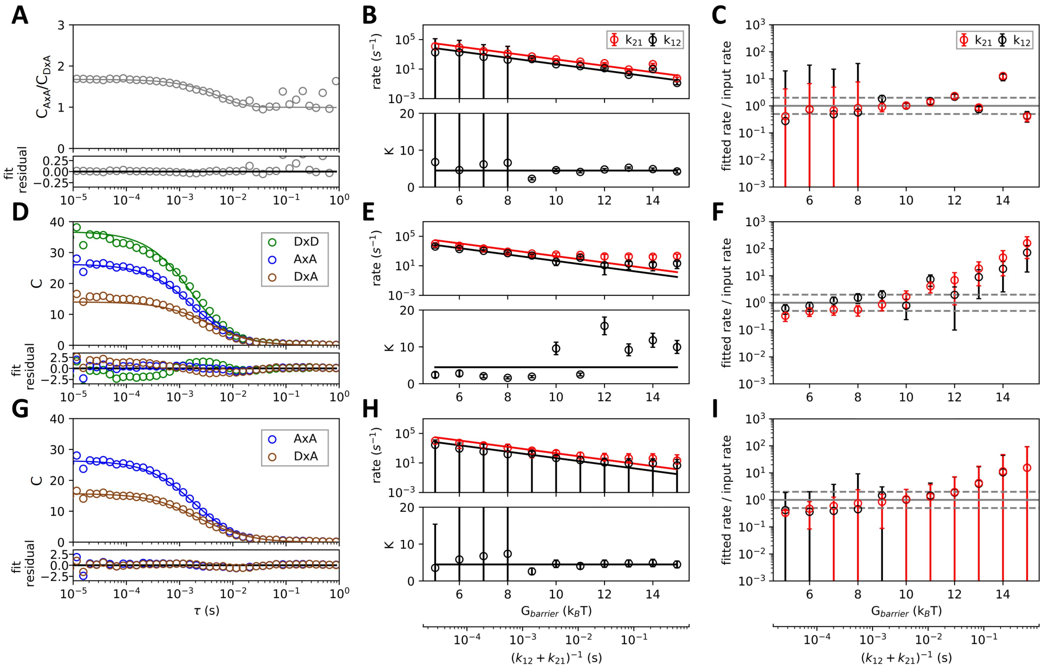

Figure S 5: **The extraction of conformation state lifetimes can be achieved through analysis of the FRET×FRET autocorrelation and non-FRET×FRET cross-correlation.** $R_1 = 10$ **nm,** $R_2 = 15$ **nm. A)** Dividing the FRET auto-correlation by the FRET×non-FRET cross-correlation divides out most contributions from diffusion. Conformational dynamics then determined the remaining function. $G_{barrier} = 10\,k_BT$. The bin size is 1 ms. **B)** Transition rates k_{12} and k_{21} (top) and equilibrium constants K (bottom) fitted with method from A) for increasing $G_{barrier}$. The bin size is 1 ms. **C)** Ratios of the fitted transition rates to their input rates from B) show slight underestimation for short lifetimes and increasing deviation at long lifetimes. Dashed lines show a factor-2 difference. **D)** Multi-correlation fit of non-FRET auto-correlation ($D \times D$, green), FRET auto-correlation ($A \times A$, blue), and non-FRET×FRET cross-correlation ($D \times A$, brown). Fit is generally poor. $G_{barrier} = 10\,k_BT$. **E)** Same as B) but for the multi-correlation fitting method from D). Fitted values for K deviate significantly from the input values. **F)** Same as C) but for the multi-correlation fitting method from D). **G)** Multi-correlation fit of FRET auto-correlation ($A \times A$, blue), and non-FRET×FRET cross-correlation ($D \times A$, brown). The accuracy of the fit is improved by leaving out the non-FRET auto-correlation. **H)** Same as B) but for the multi-correlation fitting method from G). Fitted values have similar accuracy but with larger fitting errors. **I)** Same as C) but for the multi-correlation fitting method from G). Fit constraints for all figures were determined by FRET population overlap (case I and II), as shown in figure **??**. $\Delta G = 1.5\,k_BT$ for all figures.

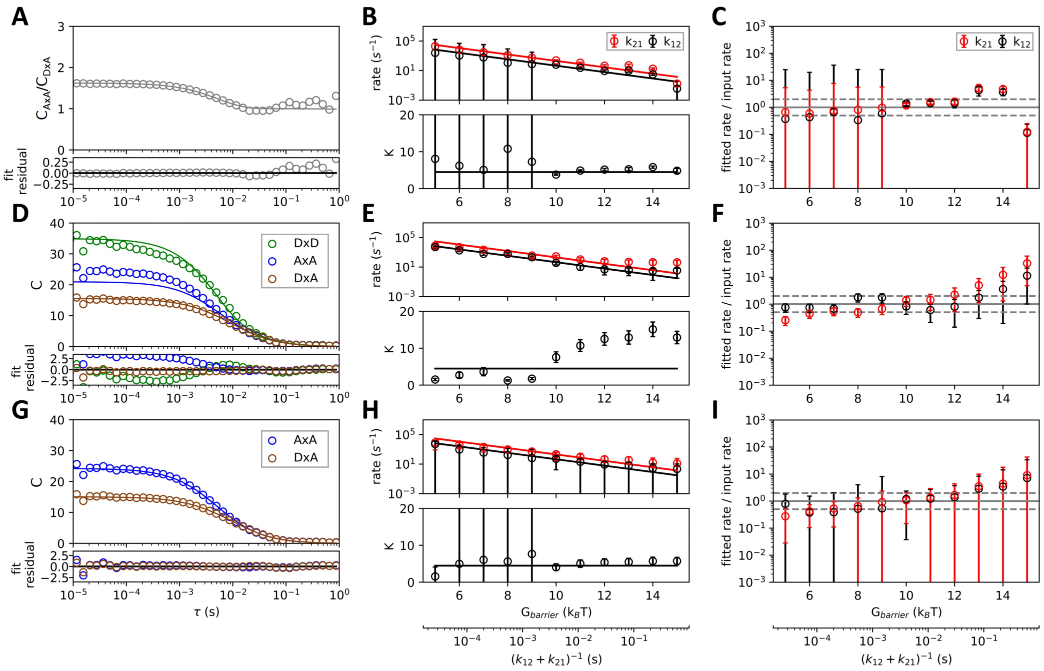

Figure S 6: **The extraction of conformation state lifetimes is not significantly altered by large differences between conformational state diffusion coefficients.** $R_1 = 30$ **nm,** $R_2 = 90$ **nm. A)-I)** Same plotted parameters and conclusions as for figure 5. $\Delta G = 1.5\, k_B T$ for all figures.

Correlation function fit parameters

Depending on the fitted function and figure, we used the following constraints:

[a] $K = f_1/f_2 = k_{21}/k_{12}$

[b] from the fitting of the histogram of burst FRET values.

[c] from the fitting of eq. **??** to the correlation function of bursts originating from only one of the FRET subpopulations.

DNA substrate

The DNA substrate used for spFRET experiments contains a Widom 601 sequence, within which two FRET labels were incorporated by PCR. The base to which the acceptor fluorophore Atto-647N was linked is highlighted in red. The position of the donor fluorophore Cy3B is highlighted in green. It was linked to a base on the complementary strand. The 601 sequence is printed in bold. Bases 60 and 61 were later found to be inverted when compared to the canonical 601 sequence. We do not expect this to have a significant influence on our results.

TGGTACGGTCTCGGGAGGA**CTGGAGAATCCCGGTGCCGAGGCCGCTCAAT-TGGTCGTAGCAAGCTCTAGCACCGCTTAAACGCACGTACGCGCTGTCCCC-**

Table 1
$C_{A \times A}$
eq. **??**
figure **??**

parameter	initial value	range [min, max]
k_{12}	10 s^{-1}	[0 , -] s^{-1}
k_{21}	100 s^{-1}	[0 , -] s^{-1}
E_1	0.8	fixed
E_2	0.1	fixed
α	0	fixed
β	1	fixed

Table 2
$C_{A \times A}$
eq. **??**
figure 2

parameter	initial value	range [min, max]
k_{12}	100 s^{-1}	[0 , -] s^{-1}
k_{21}	100 s^{-1}	[0 , -] s^{-1}
E_1	0.8	[E_2 , 1]
E_2	0.1	[0 , E_1]
α	0	fixed
β	1	fixed

Table 3
C
eq. **??**
figure 2, **??**, **??**, **??**, **??**

parameter	initial value	range [min, max]
N [a]	1/average C [b]	[0 , -]
τ_D	10^{-3} s	[0 , -] s
a	8	fixed

[a] note this value will not be accurate when correlating burst-only photons.
[b] 1/(average of C for $10^{-3} > \tau > 10^{-4}$)

Table 4
$C_{A \times A}$, $C_{D \times D}$, $C_{D \times A}$ (multi-correlation and $C_{A \times A}/C_{D \times A}$)
for $E_1 - \sigma_1 < E_2 + \sigma_2$ (case **I**)
eq. **??**-**??** + eq. **??**-**??**
figure **??**, **??**

parameter	initial value	range [min, max]
k_{12}	100 s^{-1}	[0 , -] s^{-1}
K [a]	$(E_2 - E_{overlap})/(E_{overlap} - E_1)$	fixed
$E_{overlap}$	from E hist. fit [b]	fixed
E_1	$E_{overlap} + 0.1$	[$E_{overlap}$, 1]
E_2	$E_{overlap} - 0.1$	[0, $E_{overlap}$]
$\tau_{D,1}$	10^{-3} s	[10^{-5}, 10^{0}] s
$\tau_{D,2}$	$\tau_{D,1}$	fixed

CGCGTTTTAACCGCCAAGGGGATTACTCCCTAGTCTCCAGGCACGTGTCA-GATATATACATCCTGTGACCATTGAGACCTCCGGT

Nucleosome reconstitution

Nucleosome reconstitution EMSA was run on an 0.5x TB, 0.8% agarose gel. When successfully reconstituted, nucleosomes show decreased gel shift [?]. See methods for the reconstitution protocol.

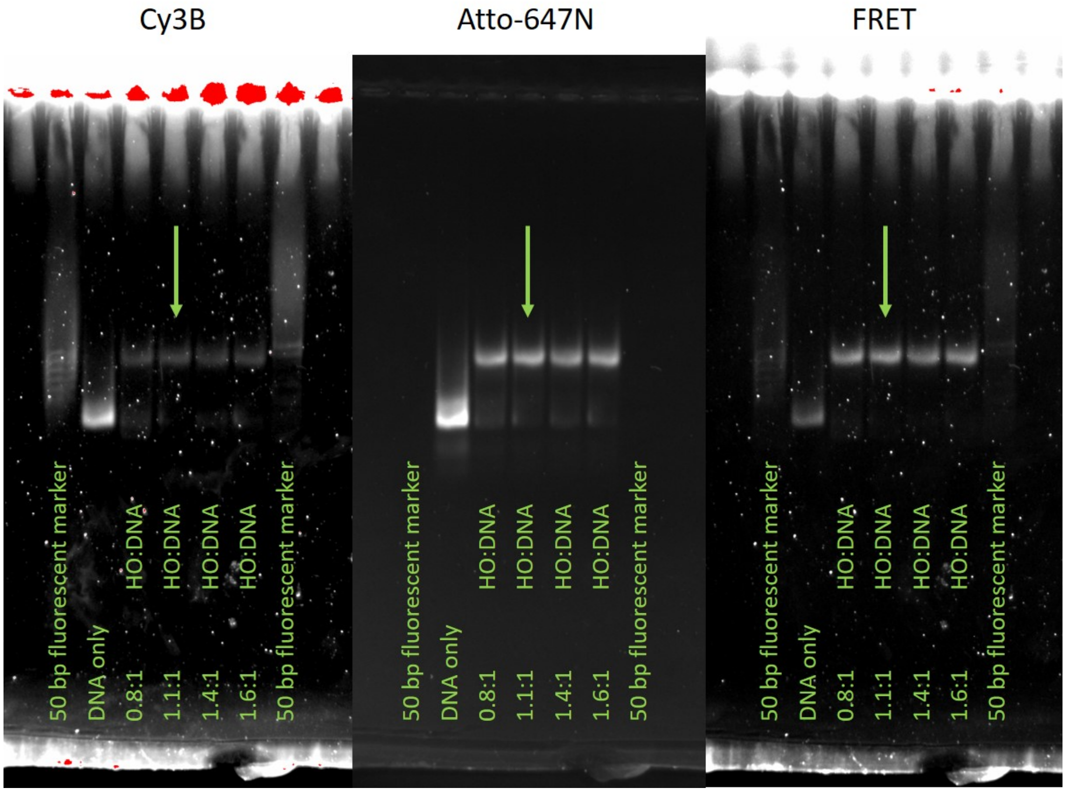

Figure S 7: **Reconstituted nucleosomes exhibit shift in agarose EMSA.** Reconstituted nucleosomes at increasing histone octamer : DNA ratio (HO:DNA) placed on gel. Donor (Cy3B), Acceptor (Atto-647N), and FRET fluorescence channels are shown. The shift relative to the DNA-only band shows reconstitution. The green arrow indicates the used sample. Gel was made with 0.5x TB, 0.8% agarose.
